# Periapical lesion-derived decellularized extracellular matrix as a potential solution for regenerative endodontics

**DOI:** 10.1093/rb/rbae050

**Published:** 2024-05-07

**Authors:** Nan Hu, Ruixue Jiang, Yuwei Deng, Weiping Li, Wentao Jiang, Ningwei Xu, Jia Wang, Jin Wen, Shensheng Gu

**Affiliations:** Department of Endodontics, Shanghai Ninth People’s Hospital, Shanghai Jiao Tong University School of Medicine, Zhizaoju Road No.639, Shanghai, 200011, China; College of Stomatology, Shanghai Jiao Tong University, Yanqiao Road No.390, Shanghai, 200125, China; National Center for Stomatology, Zhizaoju Road No.639, Shanghai, 200011, China; National Clinical Research Center for Oral Diseases, Zhizaoju Road No.639, Shanghai, 200011, China; Shanghai Key Laboratory of Stomatology, Yanqiao Road No.390, Shanghai, 200125, China; Shanghai Research Institute of Stomatology, Zhizaoju Road No.639, Shanghai, 200011, China; Department of Prosthodontics, Shanghai Ninth People’s Hospital, Shanghai Jiao Tong University School of Medicine, Zhizaoju Road No.639, Shanghai, 200011, China; College of Stomatology, Shanghai Jiao Tong University, Yanqiao Road No.390, Shanghai, 200125, China; National Center for Stomatology, Zhizaoju Road No.639, Shanghai, 200011, China; National Clinical Research Center for Oral Diseases, Zhizaoju Road No.639, Shanghai, 200011, China; Shanghai Key Laboratory of Stomatology, Yanqiao Road No.390, Shanghai, 200125, China; Shanghai Engineering Research Center of Advanced Dental Technology and Materials, Yanqiao Road No.390, Shanghai, 200125, China; Department of Prosthodontics, Shanghai Ninth People’s Hospital, Shanghai Jiao Tong University School of Medicine, Zhizaoju Road No.639, Shanghai, 200011, China; College of Stomatology, Shanghai Jiao Tong University, Yanqiao Road No.390, Shanghai, 200125, China; National Center for Stomatology, Zhizaoju Road No.639, Shanghai, 200011, China; National Clinical Research Center for Oral Diseases, Zhizaoju Road No.639, Shanghai, 200011, China; Shanghai Key Laboratory of Stomatology, Yanqiao Road No.390, Shanghai, 200125, China; Shanghai Engineering Research Center of Advanced Dental Technology and Materials, Yanqiao Road No.390, Shanghai, 200125, China; Department of Oral and Maxillofacial-Head and Neck Oncology, Shanghai Ninth People’s Hospital, Shanghai Jiao Tong University School of Medicine, Zhizaoju Road No.639, Shanghai, 200011, China; College of Stomatology, Shanghai Jiao Tong University, Yanqiao Road No.390, Shanghai, 200125, China; National Center for Stomatology, Zhizaoju Road No.639, Shanghai, 200011, China; National Clinical Research Center for Oral Diseases, Zhizaoju Road No.639, Shanghai, 200011, China; Shanghai Key Laboratory of Stomatology, Yanqiao Road No.390, Shanghai, 200125, China; Shanghai Research Institute of Stomatology, Zhizaoju Road No.639, Shanghai, 200011, China; Shanghai Center of Head and Neck Oncology Clinical and Translational Science, Zhizaoju Road No.639, Shanghai, 200011, China; Department of Endodontics, Shanghai Ninth People’s Hospital, Shanghai Jiao Tong University School of Medicine, Zhizaoju Road No.639, Shanghai, 200011, China; College of Stomatology, Shanghai Jiao Tong University, Yanqiao Road No.390, Shanghai, 200125, China; National Center for Stomatology, Zhizaoju Road No.639, Shanghai, 200011, China; National Clinical Research Center for Oral Diseases, Zhizaoju Road No.639, Shanghai, 200011, China; Shanghai Key Laboratory of Stomatology, Yanqiao Road No.390, Shanghai, 200125, China; Shanghai Research Institute of Stomatology, Zhizaoju Road No.639, Shanghai, 200011, China; Department of Endodontics, Shanghai Ninth People’s Hospital, Shanghai Jiao Tong University School of Medicine, Zhizaoju Road No.639, Shanghai, 200011, China; College of Stomatology, Shanghai Jiao Tong University, Yanqiao Road No.390, Shanghai, 200125, China; National Center for Stomatology, Zhizaoju Road No.639, Shanghai, 200011, China; National Clinical Research Center for Oral Diseases, Zhizaoju Road No.639, Shanghai, 200011, China; Shanghai Key Laboratory of Stomatology, Yanqiao Road No.390, Shanghai, 200125, China; Shanghai Research Institute of Stomatology, Zhizaoju Road No.639, Shanghai, 200011, China; Department of Endodontics, Shanghai Ninth People’s Hospital, Shanghai Jiao Tong University School of Medicine, Zhizaoju Road No.639, Shanghai, 200011, China; College of Stomatology, Shanghai Jiao Tong University, Yanqiao Road No.390, Shanghai, 200125, China; National Center for Stomatology, Zhizaoju Road No.639, Shanghai, 200011, China; National Clinical Research Center for Oral Diseases, Zhizaoju Road No.639, Shanghai, 200011, China; Shanghai Key Laboratory of Stomatology, Yanqiao Road No.390, Shanghai, 200125, China; Shanghai Research Institute of Stomatology, Zhizaoju Road No.639, Shanghai, 200011, China; Department of Prosthodontics, Shanghai Ninth People’s Hospital, Shanghai Jiao Tong University School of Medicine, Zhizaoju Road No.639, Shanghai, 200011, China; College of Stomatology, Shanghai Jiao Tong University, Yanqiao Road No.390, Shanghai, 200125, China; National Center for Stomatology, Zhizaoju Road No.639, Shanghai, 200011, China; National Clinical Research Center for Oral Diseases, Zhizaoju Road No.639, Shanghai, 200011, China; Shanghai Key Laboratory of Stomatology, Yanqiao Road No.390, Shanghai, 200125, China; Shanghai Engineering Research Center of Advanced Dental Technology and Materials, Yanqiao Road No.390, Shanghai, 200125, China; Department of Endodontics, Shanghai Ninth People’s Hospital, Shanghai Jiao Tong University School of Medicine, Zhizaoju Road No.639, Shanghai, 200011, China; College of Stomatology, Shanghai Jiao Tong University, Yanqiao Road No.390, Shanghai, 200125, China; National Center for Stomatology, Zhizaoju Road No.639, Shanghai, 200011, China; National Clinical Research Center for Oral Diseases, Zhizaoju Road No.639, Shanghai, 200011, China; Shanghai Key Laboratory of Stomatology, Yanqiao Road No.390, Shanghai, 200125, China; Shanghai Research Institute of Stomatology, Zhizaoju Road No.639, Shanghai, 200011, China

**Keywords:** decellularized extracellular matrix, pulp regeneration, periapical lesion-derived stem cells

## Abstract

Pulp regeneration remains a crucial target in the preservation of natural dentition. Using decellularized extracellular matrix is an appropriate approach to mimic natural microenvironment and facilitate tissue regeneration. In this study, we attempted to obtain decellularized extracellular matrix from periapical lesion (PL-dECM) and evaluate its bioactive effects. The decellularization process yielded translucent and viscous PL-dECM, meeting the standard requirements for decellularization efficiency. Proteomic sequencing revealed that the PL-dECM retained essential extracellular matrix components and numerous bioactive factors. The PL-dECM conditioned medium could enhance the proliferation and migration ability of periapical lesion-derived stem cells (PLDSCs) in a dose-dependent manner. Culturing PLDSCs on PL-dECM slices improved odontogenic/angiogenic ability compared to the type I collagen group. *In vivo*, the PL-dECM demonstrated a sustained supportive effect on PLDSCs and promoted odontogenic/angiogenic differentiation. Both *in vitro* and *in vivo* studies illustrated that PL-dECM served as an effective scaffold for pulp tissue engineering, providing valuable insights into PLDSCs differentiation. These findings pave avenues for the clinical application of dECM’s in situ transplantation for regenerative endodontics.

## Graphical abstract

**Figure:**
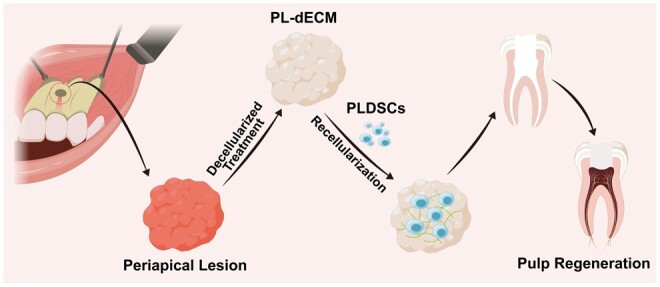

## Introduction

Dental diseases such as pulpitis and apical periodontitis, caused by bacterial infections or other factors, often lead to pain and loss of tooth tissue, presenting a troublesome problem for many individuals [1]. Currently, root canal treatment is the primary approach to address this issue, involving complete removal of the infected pulp from the root canal system and subsequent root canal filling for a better clinical prognosis [1–3]. However, endodontically treated teeth are likely to have problems such as discoloration, cracks, and fractures, highlighting the requirement to find a solution that can fully restore the tissue morphology and function of teeth [4–7]. Tissue engineering technology has provided valuable insights into regenerating pulp tissue using growth factors and biomaterials [8].

The decellularized extracellular matrix (dECM), containing plenty of bioactive factors and realistic extracellular environment, is an excellent choice for tissue engineering. These naturally derived materials undergo a special treatment to remove cellular components while retaining the extracellular matrix [9]. The dECM demonstrates exceptional biocompatibility, tissue specificity, and a porous surface structure [10, 11]. It preserves the original physicochemical microenvironment, contains cryptic peptides, and possesses the characteristics of scaffold and bioactive factors, making it an ideal environment for regenerative medicine [12, 13]. In 1948, Poel [14] first proposed obtaining decellularized homogenates from muscle samples at −70°C, leading to numerous reports on the utilization and application of decellularized treatments. For example, decellularized porcine intestinal mucosa/submucosa aids the culture of endodermal organoids and ECM scaffolds with parallel microchannels through decellularized treatment enable the directional regeneration of tissue cells [15, 16].

Similarly, recent dental research has witnessed multiple endeavors to decellularize various dental tissues, including pulp, dental follicles, and tooth germs, generating a gel-like scaffold that has been shown to promote the formation of pulp-like matrix in related animal experiments [9, 17–21]. However, the availability of autologous dental pulp or other tooth-related tissues is limited. For instance, pulp tissue can only be obtained from extracted orthodontic teeth or third molars, which falls short of a requirement to meet the high demand for regenerative scaffolds. In addition, using pulp tissue from other donors or animals raises ethical and immune issues due to the presence of heterologous antigens after decellularization, restricting its widespread clinical application [22, 23]. Therefore, there is an imperative need for a novel dECM scaffold with a comprehensive source and robust bioactivity to facilitate successful pulp regeneration.

Chronic apical periodontitis, a prevalent endodontic disease, is one period in which pulpitis progresses to the periapical tissue level, with a high prevalence rate of 52% among individuals and 5% among single tooth [3, 24]. This condition involves locally inflammatory infiltration and periapical bone destruction in the acute phase, followed by gradual restoration in the chronic phase with the assistance of immune cells, stem cells, vascular endothelial cells, etc. [25–28]. Thus, it’s evident that the microenvironment of apical periodontitis comprises various cells and bioactive factors that can either facilitate or impede tissue regeneration. Moreover, after the tooth with apical periodontitis undergoes root canal treatment to control the infection, the periapical microenvironment tends to reach a restorative state, indicating its excellent self-healing ability [29, 30]. Building upon this premise, we propose the decellularized treatment on periapical lesions from the periapical surgery to obtain the PL-dECM, which serves as a regenerative scaffold for promoting pulp regeneration and retaining some of the bioactive factors such as VEGF, VEGFR, TGF-β, FGF2, and OPN [25–28].

Furthermore, the seed cell is crucial to tissue engineering [31]. Specifically, several studies have demonstrated the promising potential of dental pulp stem cells (DPSCs) for pulp-dentin regeneration [8, 32, 33]. However, due to the limited availability of seed cells derived from oral and maxillofacial regions, periapical lesion-derived stem cells (PLDSCs) are being considered as a widely available source with mesenchymal stem cell-related antigens, good proliferation ability, and multi-directional differentiation potentials [34–37]. The mesenchymal stem cells (MSCs) from periapical lesions are prone to differentiate into osteoblast lineage cells under the stimulation of apical periodontitis [27]. Our research group has also discovered that PLDSCs exhibit less osteogenic/odontoblast differentiation ability compared to DPSCs, but they possess a more vital capability to secrete neurovascular factors and regulate the immune responses, making them potential candidates for tissue engineering of dental pulp [38, 39].

Given this context, this study investigates the possible application in pulp regeneration of dECM derived from periapical lesions (PL-dECM). We prepared the PL-dECM through decellularized treatment and analyzed its components by proteomics. Then, we cultured PLDSCs in this dECM scaffold, confirming the potential of PL-dECM on promoting pulp regeneration. The results observed from both *in vitro* and *in vivo* attempts were consistent, providing valuable insights into the future clinical utilization of autologous transplantation for facilitating pulp regeneration.

## Materials and method

### Fabrication of decellularized extracellular matrix derived from periapical lesions (PL-dECM)

#### Sample collection

The experimental process was approved by the Ethics Committee, Shanghai Ninth People’s Hospital (SH9H-2021-T112-1 and SH9H-2023-A328-SB), and the research methods followed the ethical principles of the Declaration of Helsinki. Fifteen patients aged between 18–45 years old, diagnosed with chronic apical periodontitis (PAI index = 4/5), underwent complete root canal treatments and planned to require periapical microsurgery, were recruited. These patients had no underlying systemic diseases and no recent history of medication. Before the surgery, these patients underwent relevant pre-operative examinations and provided informed consent. The specialist and surgical method remained consistent for all patients. The removed periapical lesions were divided into two parts. One was placed in sterile high-glucose Dulbecco’s Modified Eagle Medium (DMEM) and immediately stored in a −80°C refrigerator for subsequent decellularized treatment. Another part of the periapical lesion was placed in a sterile DMEM medium in a 4°C refrigerator for cell culture.

#### Decellularized treatment

(i) the periapical lesion tissue was rinsed with sterile PBS three times for 3 min each time; (ii) the tissue was placed in a sterile 0.2% sodium dodecylbenzene sulfonate solution (0.2% SDS solution, volume ratio is ∼1:20) and subjected to agitation on a reciprocating shaker at 15 rpm for 12 h, maintaining the ambient temperature of 4°C; (iii) the second step was repeated, followed by immersing the sample in sterile double-distilled water three times for one hour each time; (iv) the sample was treated with 50 U/ml DNase solution at 37°C for 1 h; (v) Subsequently, the sample was soaked in sterile double-distilled water three times for 2 h each time; (vi) finally, the dECM was stored in PBS solution, and placed in a −80°C refrigerator.

### Isolation and culture of PLDSCs

The periapical lesions were thoroughly rinsed with sterile PBS containing 100 U/ml penicillin and 0.1 mg/ml streptomycin to prepare primary outgrowth cultures. Then, the periapical lesions were cut into small parts (∼1 mm in diameter) using sterile surgical scissors. The small pieces of tissue were transferred to a petri dish and covered with a sterile coverslip fixed in sterile vaseline. Cells were cultured using DMEM supplemented with 20% fetal bovine serum, 100 U/ml penicillin and 0.1 mg/ml streptomycin. The petri dish containing the primary outgrowth was placed at 37°C with 95% air and 5% carbon dioxide. The culture medium of primary outgrowth cultures was replaced every 3 days, and the cultured cells were passed to a new petri dish when reaching nearly confluence using DMEM supplemented with 10% fetal bovine serum, 100 U/ml penicillin and 0.1 mg/ml streptomycin.

### Quantitative assays of the periapical lesion and decellularized extracellular matrix (DNA, collagen and glycosaminoglycan)

#### DNA extraction, quantification and electrophoresis

The weights of the periapical lesion and PL-dECM were recorded under dry conditions after dehydration. DNA was extracted, separated and purified using a DNA extraction kit (DP304, TIANGEN, China) according to the instructions. The remaining DNA was quantified using a spectrophotometer. The experimental processes were conducted three times, and the DNA contents of the periapical lesion and PL-dECM were normalized based on sample mass (ng/mg). Gel electrophoresis was obtained using a 1% agarose gel in TAE buffer. Markers and DNA samples with loading buffer were added to the lanes, and the gel was then run at 100 V in a gel-electrophoretic apparatus until the dye line reached ∼75–80% of the length of the gel. The resulting image was visualized using a gel imager system.

#### Collagen quantification

According to the steps of the Hydroxyproline (HYP) Content Assay Kit (BC0255, Solarbio, China), the samples were added to the extraction solution and treated at 100°C for 2 h. The pH of the solution was adjusted to neutral using alkaline liquid, and the supernatants from different samples were collected after centrifugation. The concentrations of hydroxyproline in the samples were further determined according to the kit protocol, and the hydroxyproline contents of the periapical lesion and PL-dECM were normalized by sample mass (μg/mg).

#### Glycosaminoglycan quantification

The samples were immersed in a cooled PBS solution and made tissue homogenates. The supernatants were collected after centrifugation. The ELISA was performed according to the kit protocol (Human GAG ELISA Kit, EH0988, BIOLOSINE, China). The absorbances at 450 nm were orderly recorded and the glycosaminoglycan (GAG) contents in the periapical lesion and PL-dECM were calculated and normalized based on the standard curve and sample mass (ng/mg).

### Scanning electron microscopy

The periapical lesion and PL-dECM were rinsed with PBS, fixed with 3% glutaraldehyde solution at 4°C overnight, and dehydrated using ethanol gradient elution. The samples were dried, sputter-coated with gold, and observed using a scanning electron microscope (SU8010, Hitachi, Japan).

### Histological staining

The periapical lesion and PL-dECM were fixed with 4% paraformaldehyde at 4°C for 24 h, rinsed with running water, dehydrated with gradient ethanol, transparent in xylene, soaked with paraffin, embedded and cut into 4-μm sections. The histologic sections of the periapical lesion and PL-dECM underwent H&E staining for observation of general structure, Masson’s trichrome staining to observe collagen distribution, Sirus red staining to observe specific collagen type and Safranin-O staining to identify the GAG distribution. The main components of the extracellular matrix in the periapical lesion and PL-dECM were identified through immunofluorescence staining, using antibodies listed in Supplementary Table 3.

### Proteomic analysis

dECM samples from periapical lesions (*n* = 3) were analyzed by 4D data-independent acquisition quantity proteomics. Specifically, the extracted proteins were digested with 0.25% trypsin-EDTA, desalinized, detected by LC-MS/MS high-resolution mass spectrometry, and then processed with the raw DIA data by Spectronaut Pulsar 17.5. The protein functions were categorized using Gene Ontology annotation provided by https://david.ncifcrf.gov/. Bar charts were used for visualization. MatrisomeDB 2.0, an ECM-specific classification database, was applied to evaluate the characteristics of the PL-dECM samples [40].

### Measurements of cell viability and migration ability

#### Preparation of culture medium

The PL-dECM was lyophilized and its dry weight was measured and marked. The PL-dECM leachate with a concentration of 1 mg/ml was prepared by adding 1 ml DMEM to 1 mg PL-dECM lyophilizate on a shaker for 24 h at 37°C. Referring to the protocol from Alqahtani *et al.* [18], every 1 mg dry weight of PL-dECM was digested in 50 μl 1 mg/ml pepsin (pH = 2, P8160, Solarbio, China) and was placed on a shaker for 48 h at 37°C. Then, the solution was neutralized with sterile 0.1 mol/l NaOH solution, and DMEM solution was added to achieve a concentration of 1 mg/ml in the PL-dECM conditioned medium.

#### Cell viability

The third passage and well-grown PLDSCs were seeded in 96-well plates at a density of 5 × 10^3^ cells per well, and different concentrations of PL-dECM leachate or PL-dECM conditioned medium were applied the next day. CCK-8 solution (CK04, Dojindo, Japan) was added to each well after culturing for 1, 3 and 7 days and incubated for an hour at 37°C. The absorbances were determined at 450 nm using a microplate reader. The relative cell viability of different groups at different time points was determined by comparing its absorbance to that of the control group on the first day, and histograms were used for visualization.

#### Migration ability test

For the transwell migration assay, PLDSCs with serum-free DMEM were added to the upper compartment of the transwell insert (3422, Corning, USA) at a concentration of 2.5 × 10^4^/ml, while the lower compartment was filled with different concentrations of PL-dECM leachate or PL-dECM conditional medium containing 1% FBS at the same time. After culturing at 37°C and 5% CO_2_ for 12 h, the transwell inserts were incubated with 4% paraformaldehyde for 20 min and stained with 1% crystal violet solution (G1062, Solarbio, China) for 15 min. Then, PLDSCs on the upper part of the microporous membrane were carefully wiped off with cotton swabs. PLDSCs which successfully crossed the membrane were photographed under an inverted phase contrast microscope. The cell numbers were counted by ImageJ, and a summary table was generated. For the wound healing assay, when PLDSCs in the 24-well plate reached confluence, the cells were starved for 24 h to exclude the interference of proliferation with the results. After scraping the cell monolayer along a straight line, the cells in different wells were cultured with varying concentrations of PL-dECM leachate or PL-dECM conditioned medium at 37°C and 5% CO_2_ for 12 h. At the time points of scraping and culturing for 12 h, Hoechst staining solution (C1027, Beyotime, China) for live cell staining was used to visualize the changes in cell migration with the aid of fluorescence microscope. The area of migrated cells was counted using ImageJ.

### Quantitative RT-PCR

To evaluate the effect of PL-dECM on PLDSCs, 100 μm thick slices of PL-dECM were used as a culture substrate, with type I collagen as a control group. The cells were cultured on either PL-dECM slices or type I collagen, respectively. Then, total RNA samples from different groups were extracted with TRIzol Reagent (Invitrogen, USA) and reverse transcribed into cDNA using a cDNA Synthesis Kit (11119ES60, Yearson, China) after 3 and 7 days of culture. Quantitative reverse transcription PCR was performed to observe the gene expressions at the RNA level under the following method: first 95°C for 30 s, then 40 conversional cycles of 95°C for 10 s and 60°C for 30 s. The gene expression was calculated based on the relative CT value compared to the housekeeping gene expression. The specific primer sequences are shown in Supplementary Table 1, commercially synthesized by Sangon Biotech Shanghai Co Ltd.

### Western blot

For the periapical lesion and its PL-dECM, appropriate amounts of RIPA lysis buffer (P0013B, Beyotime, China) with PMSF (ST505, Beyotime, China) and protease and phosphatase inhibitors (P1048, Beyotime, China) were added to extract the total content of the tissue with the assistance of ultrasound. The supernatant was collected for further protein analysis after centrifuging at 15 000 rpm for 15 min at 4°C. Subsequently, the sample concentration was consistently adjusted according to the bicinchoninic acid assay (P0012S, Beyotime, China) to ensure equal protein amounts. The samples were utilized in electrophoresis using ready-made SDS-PAGE gel (M42012C, GenScript, China). The gels were stained with Coomassie Brilliant Blue staining solution (P0017F, Beyotime, China) for 15 min, washed with double-distilled water, and photographed.

For cell samples, the same method was implemented to extract total protein. The cell lysates of equal concentration and volume were separated by electrophoresis using pre-made SDS-PAGE gel (M41215C, GenScript, China). The protein samples on the SDS-PAGE gel were then transferred to 0.22 μm PVDF membrane (GVWP04700, Millipore, USA). After blocking with 5% non-fat milk, the protein bands were incubated with primary antibodies at 4°C overnight. Rinsing the protein bands with TBST (PS103S, Epizymes, China) and then incubating them with HRP-coupled secondary antibodies at room temperature for 1 h. ECL Western Blot Reagent (WBULS0100, Millipore, USA) was used for development, and images were recorded with a gel image system. Details of primary and HRP-coupled secondary antibodies are provided in Supplementary Table 2.

### Live/dead cell viability assays

The PLDSCs were seeded onto microscope cover glasses (801010, NEST, China), coated with type I collagen or PL-dECM slices, at a density of 1 × 10^5^/ml. On the 3rd and 7th days of culture, the cells were incubated by Live/Dead Viability Assay Kit (C2015S, Beyotime, China) at 37°C under dark conditions for 30 min. Then, the cellular ability was observed and recorded using a fluorescence microscope with wavelengths of 490 nm and 545 nm.

### Immunocytochemistry staining

Similarly, PLDSCs were seeded on confocal dishes (BS-15-GJM, Biosharp, China), coated with type I collagen or PL-dECM slices at a density of 2 × 10^4^/ml. The PLDSCs culturing for three days or seven days were fixed with 4% paraformaldehyde for 20 min, treated with 3% BSA and 0.1% Triton-X buffer for 30 min, incubated with primary antibodies at 4°C overnight, and treated with corresponding secondary antibodies at room temperature for 1 h in the dark conditions. The nuclei were stained with DAPI staining solution (C0065, Solarbio, China) for 5 min. The final staining results were observed and recorded by a confocal laser microscopy. The primary and secondary antibodies are presented in Supplementary Table 3.

### *In vivo* transplantation of tooth section

Six-week-old BALB/c nude mice were obtained from the Animal Center of the Ninth People’s Hospital Affiliated to Shanghai Jiao Tong University School of Medicine, with approval of all procedures from the Institutional Animal Care and Use Committee (SH9H-2021-T112-1 and SH9H-2023-A328-SB). Healthy premolars extracted from the hospital for orthodontic needs were collected. The premolars were processed into tooth sections with a length of 5 mm, and their periodontal tissue, cementum and pulp tissue were removed simultaneously. The tooth sections were treated with 17% EDTA solution for 5 min, ultrasonically cleaned for 10 min, sterilized by soaking in PBS solution containing the Penicillin-Streptomycin-Amphotericin B for 48 h and stored in sterile PBS solution for later use. The experiment was designed as follows: (i) the experimental group, PL-dECM scaffold with 1 × 10^6^ PLDSCs; (ii) the control group, type I collagen with 1 × 10^6^ PLDSCs. Two groups of scaffolds were placed in the middle cavity of the tooth segment. The nude mice were anesthetized via intraperitoneal injection of dexmedetomidine and Zoletil in a volume of 0.1 ml. Incisions were made bilaterally on the mid-dorsal region, followed by blunt dissection. The experimental and control groups were implanted in the left and right sides, respectively. Sham-operated mice underwent dorsal incisions without tooth section implantation. After 8 weeks, the mice were euthanized for sample collection. The samples were fixed with 4% PFA for 24 h, decalcified with 10% EDTA solution for 12 weeks (the decalcification solution was changed every two days), and processed through gradient ethanol dehydration, xylene permeabilization, embedding and sectioning. H&E and immunohistochemical staining were performed for observation.

The immunohistochemical staining followed a similar protocol to immunofluorescence staining. The sample sections were first deparaffinized in xylene, rehydrated in gradient ethanol, treated with pepsin for antigen retrieval, inactivated endogenous enzymes with 3% hydrogen peroxide solution, blocked with 3% BSA-PBS, then incubated with primary antibodies at 4°C overnight, treated with HRP-coupled secondary antibody for 1 h, and finally treated with DAB solution. The sample sections were observed under a microscope after the nuclei were stained with hematoxylin staining. Additionally, when the nude mice were euthanized, their essential organs, the liver, spleen, lung and kidney, were also collected and made into tissue sections. The sections underwent H&E staining to evaluate the impact of the transplants compared with the sham-operated group. Detailed antibody information is listed in Supplementary Table 3.

### Statistical analysis

The experiments were performed independently at least three times. After passing the Normality Test, the data were analyzed through the Student’s *t*-test or one-way analysis of variance in GraphPad Prism 9.0 software. The *P* value <0.05 was considered statistically significant.

## Result

### Fabrication and characteristics of decellularized extracellular matrix from periapical lesions (PL-dECM)

**Figure 1.**
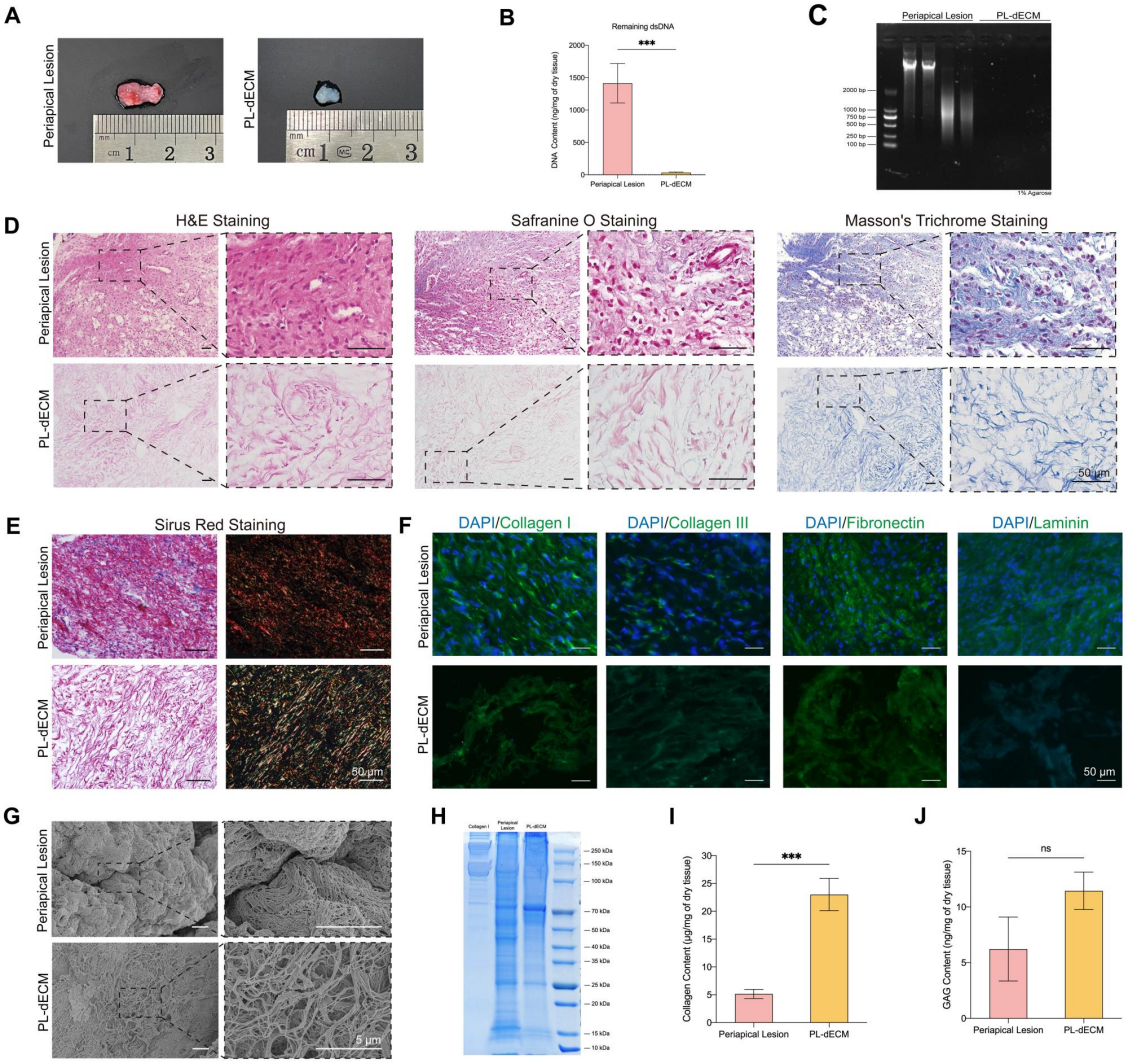
The preparation and characteristic of PL-dECM. (**A**) Gross observation image showed correlations and differences between the periapical lesion and PL-dECM. (**B**) DNA quantitative detection displayed that the DNA content of PL-dECM was significantly reduced than the periapical lesion (***: *P* < 0.001). (**C**) DNA gel electrophoresis of periapical lesion and PL-dECM presented that there is basically no DNA component after decellularized treatment. (**D**) H&E, Safranine O and Masson’s trichrome staining described basic structural changes of the periapical lesion and PL-dECM. (**E**) Observations under brightfield and polarizer field of Sirius red staining revealed the expression of collagen subtypes in the periapical lesion and PL-dECM. (**F**) Immunofluorescence staining of the main components of the extracellular matrix in the periapical lesion and PL-dECM. (**G**) Scanning electron microscope images showed that decellularized treatment caused significant changes from a microscopic perspective. (**H**) Coomassie brilliant blue staining showed the differences of protein expression between type I collagen, periapical lesion and PL-dECM. (**I**, **J**) The quantification of collagen and glycosaminoglycan in the periapical lesion and PL-dECM (***: *P* < 0.001, ns: not significant); scale bar: 50 μm (histological staining); 5 μm (SEM).

As shown in the Graphic Abstract, periapical lesions obtained from periapical surgery were subjected to decellularized treatment to produce PL-dECM. PLDSCs were also isolated and cultured to combine with the PL-dECM for tissue regeneration. Referring to a method described by Alqahtani *et al.* [18], we successfully fabricated dECM from periapical lesions. The resulting PL-dECM appeared as a volume-reduced, transparent, colorless and amorphous gelatinous material, in contrast to the pink and soft periapical lesions observed grossly (Figure 1A).

To assess the efficiency of decellularization, DNA quantification assay and gel electrophoresis of the periapical lesion and PL-dECM were performed. It was found that the remaining dsDNA in the PL-dECM was <50 ng/mg of dry tissue, which is significantly lower than that of periapical lesions. The PL-dECM showed almost no fluorescent band, while the periapical lesion appeared with distinct DNA segments in gel electrophoresis, indicating the absence of DNA in the PL-dECM (Figure 1B and C). Histological analysis revealed a lack of hematoxylin-stained nuclear structure but the presence of eosin-colored wavy structures in the tissue sections of PL-dECM, showing a contrast between the nucleus and cytoplasm structures in the periapical lesion (Figure 1D). The above results met the safety standards for decellularized tissue proposed by Crapo *et al.* [41], demonstrating the feasibility of the method.

Furthermore, the presence of GAG in the PL-dECM was confirmed through Safranin O staining. Masson’s trichrome staining revealed that it had a blue-purple stained nucleus and a large amount of blue-stained collagen fibers in the periapical lesion, while looser blue-stained collagen fibers were observed in the PL-dECM (Figure 1D). To examine the condition of collagen fibers, paraffin sections of both samples were stained with Sirius Red Stain Kit and observed using a polarizing microscope, which showed that the periapical lesion contained plenty of closely arranged and red-stained type I collagen, whereas the PL-dECM mainly included red- or yellow-stained type I collagen and green-stained type III collagen (Figure 1E). Immunofluorescence staining also confirmed the presence of type I collagen, type III collagen, fibronectin and laminin, which are the main components of the pulp extracellular matrix, in both the periapical lesion and its PL-dECM scaffolds (Figure 1F). Despite a weaker fluorescence intensity compared to the sample before decellularization, these results did demonstrate that PL-dECM’s similar extracellular matrix composition as the periapical lesion.

Microstructural analysis revealed that the periapical lesion exhibited a structure in which local cells were wrapped in thin and tightly wound fibers, while the PL-dECM showed a relatively loose and thin fibrous structure without any observed cell-like structures. After the decellularization treatment, the microstructure of the extracellular matrix was partially preserved, facilitating subsequent recellularization (Figure 1G). Coomassie brilliant blue staining visualized that PL-dECM retained a portion of the protein components after decellularized treatment (Figure 1H). Hydroxyproline quantification showed that PL-dECM was rich in collagen, with a content of 22 μg/mg in dry weight, approximately four times higher than that of the periapical lesion (Figure 1I). The GAG contents were similar in both samples as detected by the ELISA kit (Figure 1J). The measured samples were dehydrated as instructed by the quantitative methods. Due to the higher water content and the relatively higher extracellular matrix distribution after dehydration treatment, the PL-dECM showed a more considerable amount of collagen and GAG contents per unit dry weight compared to periapical lesions. The spatial distribution and relative content of wet tissues were shown in Masson’s trichrome staining and Safranine O staining—the collagen fibers and GAGs in the PL-dECM were lower than those in periapical lesions. These results illustrated the obtained PL-dECM met the decellularized requirements, maintained the main components of the ECM, and had a loose and porous microstructure, suggesting its potential as a regenerative scaffold with favorable cell affinity.

### Proteomic analysis of PL-dECM

**Figure 2.**
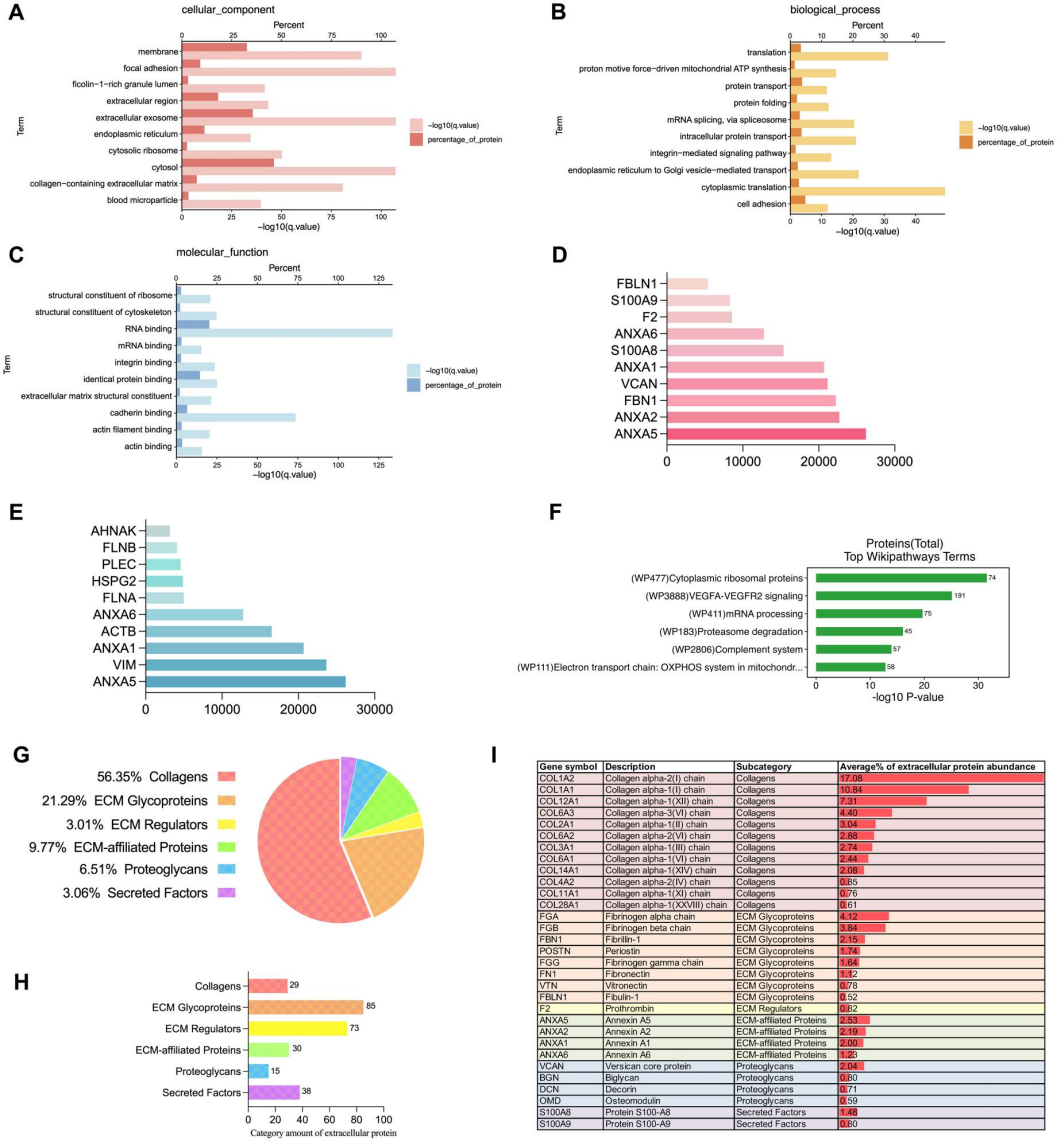
Proteomic analysis of the PL-dECM. (**A**–**C**) GO enrichment analysis showed the protein enrichment degree of cellular components, biological process, and molecular function of the PL-dECM. (**D**, **E**) The top 10 highly expressed proteins in the calcium-binding and focal adhesion categories of GO analysis. (**F**) Wikipathways analysis revealing the most relevant pathways in the PL-dECM components. (**G**, **H**) The MatrisomeDB 2.0 database was used to analyze the relative content and category amount of extracellular matrix proteins in the PL-dECM. (**I**) The graph exhibited the relatively highly expressed proteins and their relative contents in different categories of extracellular matrix proteins.

After assessing the quality of the dECM derived from the periapical lesion (*n* = 3), 4D-DIA proteomic sequencing showed that the PL-dECM contained 3076 proteins and 23 431 peptides, and proteomics was then applied to analyze the composition and function of PL-dECM. Gene Ontology enrichment analysis indicated that cellular components such as the membrane, extracellular matrix-related ingredients and adhesion-related proteins were abundant. Regarding biological processes and molecular functions, the PL-dECM was closely associated with protein transport, translation, and cell adhesion (Figure 2A–C). Further analysis of calcium binding and focal adhesion indicated an abundance of annexins family and cytoskeleton-related proteins, which positively regulate the osteogenic differentiation of MSCs (Figure 2D and E). Wiki pathways analysis exhibited that the PL-dECM components are more related to protein synthesis-related pathways, VEGFA-VEGFR2 pathway, complement pathway and cell metabolism pathway, suggesting their potential to promote cellular protein synthesis and angiogenesis (Figure 2F).

Then, the ECM-specific classification database (MatrisomeDB 2.03 [42]) was used to analyze the characteristics of PL-dECM, revealing a high abundance of extracellular matrix proteins, with collagen representing the majority (56.35%), followed by glycoproteins, ECM-affiliated proteins, proteoglycans, ECM regulatory proteins and secreted factors (Figure 2G and H). The abundant proteins in each subclass are enumerated in Figure 2I, indicating a specific regeneration microenvironment formed by various extracellular matrix proteins in the PL-dECM. The top 100 expressed proteins are listed in Supplementary Table 4.

### Bioactive effect of PL-dECM *in vitro*

#### The isolation, culture, and characterization of PLDSCs

To comprehensively evaluate the properties of PL-dECM *in vitro*, PLDSCs were chosen as the target for *in vitro* experiments. PLDSCs were isolated and cultured from the periapical lesion (Supplementary Figure S1A). The growth curve plotted by CCK-8 detection and colony-forming assay demonstrated the great proliferation ability of PLDSCs (Supplementary Figure S1B and C). These cells possessed positive expression of MSC-associated surface antigens as CD90 (98.7%), CD105 (95.7%) and CD73 (81.7%), while showing merely no expression of surface markers on hematopoietic stem cells, CD20, CD31 and CD34 (all < 0.25%), through flow cytometry analysis (Supplementary Figure S1D). The alkaline phosphatase staining of PLDSCs deepened after osteogenic culture for 3 and 7 days, and Alizarin red staining showed red-stained calcium nodules formed after 21 days of osteogenic induction (Supplementary Figure S1E and F). Similarly, PLDSCs showed significant increases in osteogenesis/odontogenesis-related markers at the mRNA and protein levels after 7 and 14 days of osteogenic induction (Supplementary Figure S1G and H). Therefore, since PLDSCs are relatively easy to obtain and have good proliferation and odontogenic/osteogenic differentiation capabilities, PLDSCs were used to evaluate the viability of PL-dECM *in vitro*.

#### Effects of PL-dECM-related medium on PLDSCs

**Figure 3.**
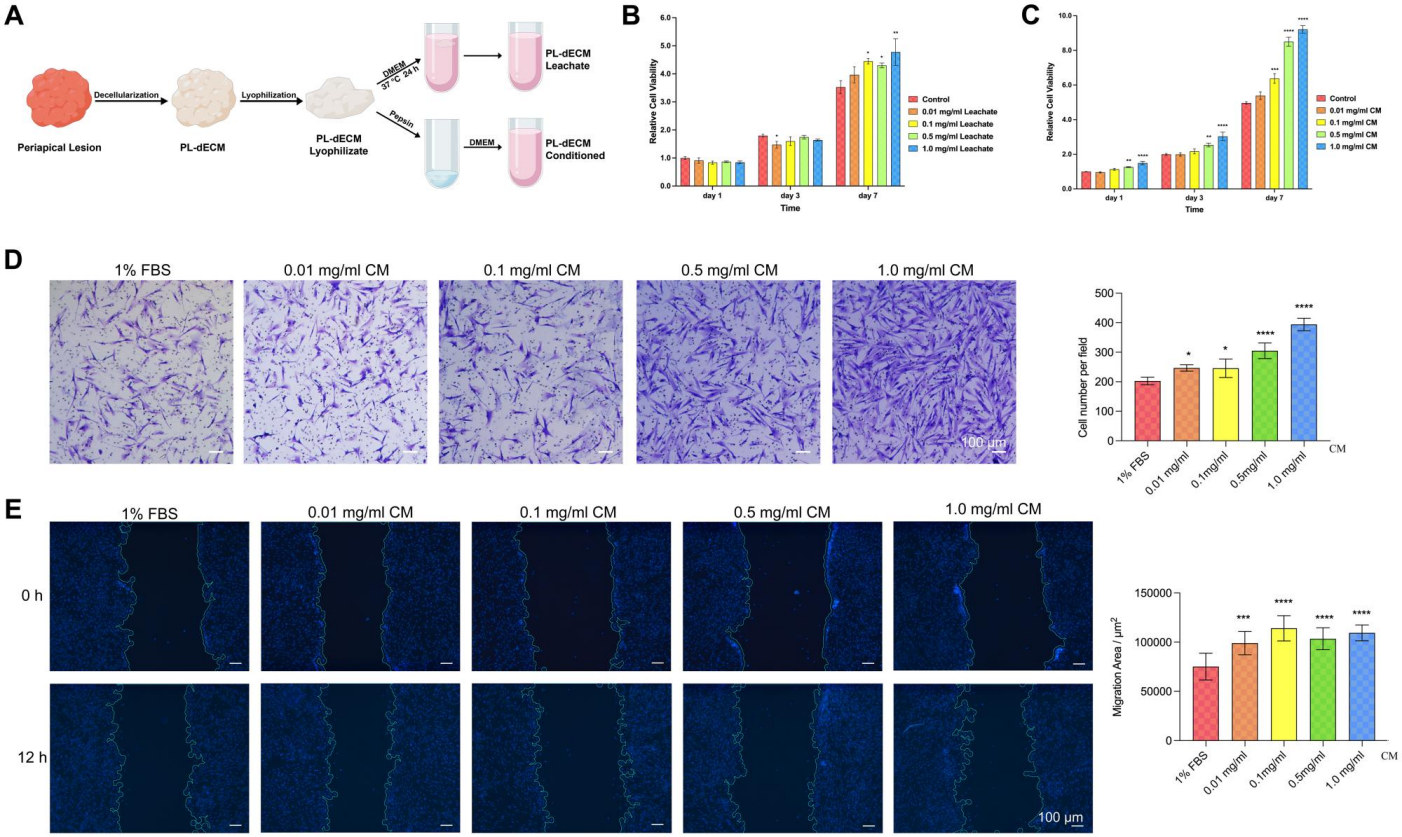
Bioactive effects of PL-dECM *in vitro*. (**A**) Schematic of the preparation of PL-dECM leachate and conditioned medium; (**B**, **C**) the effects of PL-dECM leachate (B) and conditioned medium (C) on the proliferation ability of PLDSCs (*: *P* < 0.05, **: *P* < 0.01, ***: *P* < 0.001, ****: *P* < 0.0001 as compared with the control group). (**D**, **E**) Transwell assay (D) and wound healing assay (E) demonstrated the promoting effect of PL-dECM conditioned medium on the cellular migration ability (*:*P* < 0.05, ***: *P* < 0.001, ****: *P* < 0.0001 as compared with the 1% FBS group); leachate: PL-dECM leachate; CM: PL-dECM conditioned medium; scale bar: 100 μm.

The PL-dECM leachate and PL-dECM conditioned medium were prepared as described in the illustration (Figure 3A) and gross photography (Supplementary Figure S2). The CCK-8 assay showed that PL-dECM leachate had little effect on cell growth after culturing PLDSCs with different leachate concentrations for 1, 3 and 7 days (Figure 3B). Similarly, the migration ability of PLDSCs was hardly affected by PL-dECM leachate through transwell migration assay and wound healing assay (Supplementary Figure S3). However, PLDSCs exhibited enhanced proliferation ability when co-cultured with PL-dECM conditioned medium for 1, 3 and 7 days. As the concentration of PL-dECM conditioned medium changed from 0.01, 0.1, 0.5 and to 1.0 mg/ml, the proliferation ability of PLDSCs gradually improved (Figure 3C). Transwell migration assay demonstrated that PL-dECM conditioned medium promoted the migration ability of PLDSCs. The number of cells migrating to the lower compartment increased with the increasing concentration of PL-dECM conditioned medium, as indicated by ImageJ analysis (Figure 3D). The dose-dependent effect of PL-dECM conditioned medium on promoting the migration ability of PLDSCs was also observed in the wound healing assay (Figure 3E). The surprising impact of PL-dECM conditioned medium proved its nutritious property, indicating that the active ingredients released from the PL-dECM, accompanied by the process of cellular adhesion and extracellular matrix reconstruction, have robust and positive effects on cellular behaviors.

#### Effects of PL-dECM slices on PLDSCs

**Figure 4.**
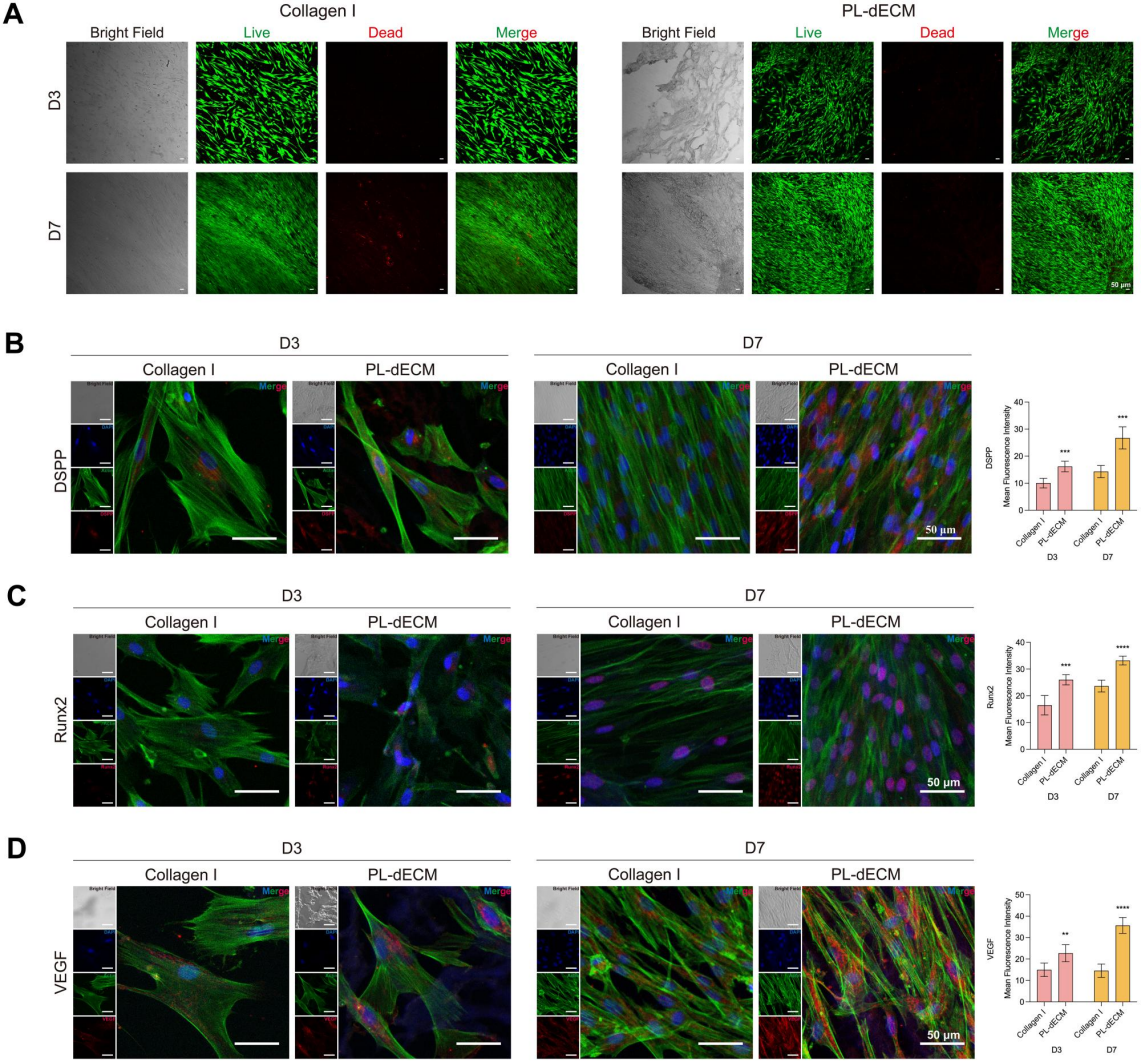
PL-dECM slices for PLDSCs culture. (**A**) The viability of PLDSCs co-cultured with type I collagen or PL-dECM slices was observed under light microscopy and fluorescence microscope after staining by live/dead viability assay. (**B**–**D**) Immunocytochemistry staining revealed the protein expression of DSPP, Runx2 and VEGF in PLDSCs co-cultured with type I collagen or PL-dECM slices (**: *P* < 0.01, ***: *P* < 0.001, ****: *P* < 0.0001 as compared with the type I collagen group); scale bar: 50 μm.

To further evaluate the *in vitro* effect of the PL-dECM, dECM derived from the periapical lesion was processed into 100-μm thick slices for the cultivation of PLDSCs, with type I collagen as the control group (Supplementary Figure S4). As shown in Figure 4A, the morphology of PLDSCs cultured on PL-dECM slices exhibited close attachment to the fibers, aligning approximately with the fiber course, which was a little messier than the control group. PLDSCs in the type I collagen group grew in a common direction. The viability of cells cultured on PL-dECM slices or type I collagen was visualized using the Live/Dead Viability Assay Kit, which showed that both groups had equivalent numbers of viable cells with minimal cell death on the third day of co-culture. After culturing for 7 days, cells in both groups were tightly packed, with a small number of dead cells observed in the type I collagen group. The cells on the PL-dECM slices demonstrated a more orderly arrangement along the fibers in three-dimensional space, with almost no PI-stained dead cells.

Immunocytochemistry staining confirmed that PL-dECM positively influences cell differentiation, and PLDSCs cultured on PL-dECM slices or type I collagen exhibited better morphology with distinct nuclei stained by DAPI and F-actin stained by Phalloidin-FITC (Figure 4B–D). On the third day of culture, PLDSCs in the type I collagen group displayed a larger cell volume than the PL-dECM group and were arranged as spindle-shaped on the seventh day. In addition, the red fluorescently labeled DSPP, Runx2 and VEGF of PLDSCs in the PL-dECM slice group displayed higher expression levels than the type I collagen group after co-cultured for 3 and 7 days, suggesting that PL-dECM provides better guidance for the odontogenic and angiogenic differentiation. Simultaneously, RNA and protein samples collected from PLDSCs cultured on PL-dECM slices or type I collagen for 3 and 7 days were extracted. It showed significant improvement in the expression of odontogenic/angiogenic-related markers, DSPP, DMP-1, Runx2 and VEGF, on mRNA and protein levels compared to the control group through RT-qPCR and western blot analysis (Supplementary Figure S5).

In addition, degradation experiments were performed to further evaluate the characteristics of type I collagen and PL-dECM. During the hydrolytic degradation test, it was observed that type I collagen gradually shrank to an amorphous structure with its weight decreased to ∼4.39% of the original one by the seventh day. In contrast, the PL-dECM maintained a translucent and gel-like structure. Its weight reduced to ∼78.04% compared with the original weight, possibly due to a decrease in water-holding capacity (Supplementary Figure S6A and D). In the enzymatic degradation test, the type I collagen and PL-dECM showed different results. The type I collagen displayed significant susceptibility to degradation, being completely degraded after 60 min of collagenase treatment and experiencing a mass reduction to 4.94% of its original value after 120 min of dispase treatment. However, the PL-dECM demonstrated only partial degradation during collagenase test, with its mass reduced by half. The PL-dECM maintained its original structure relatively well during dispase treatment, with a mass reduction of ∼37.81% after 120 min of treatment (Supplementary Figure S6).

### Subcutaneous transplantation model to evaluate the ability to promote pulp regeneration of PL-dECM *in vivo*

**Figure 5.**
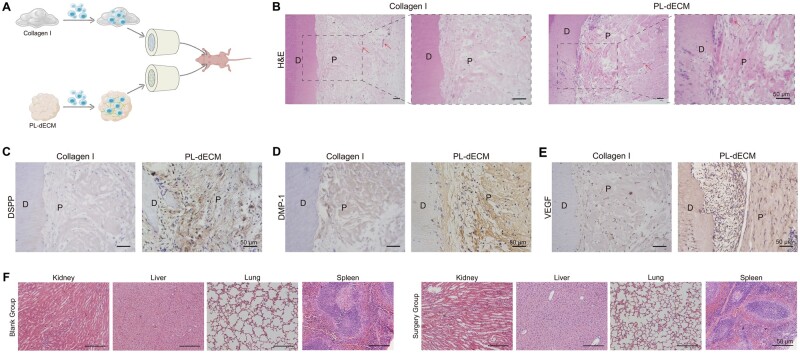
Subcutaneous transplantation model to evaluate the effect of PL-dECM. (**A**) Schematic to explain experimental design of subcutaneous transplantation. (**B**–**E**) H&E and immunohistochemical staining results on type I collagen group and PL-dECM group *in vivo*; arrow: blood vessels; D, dentin; P, pulp. (F) H&E staining to assess the toxicity of subcutaneous graft; scale bar: 50 μm.

A subcutaneous transplantation model was constructed in nude mice to assess the bioactive roles of PL-dECM *in vivo*. Tooth sections loaded with either type I collagen (control group) or PL-dECM (experimental group) along with PLDSCs were implanted into the subcutaneous area of nude mice, as illustrated in Figure 5A. After 8 weeks, the tooth sections were harvested, processed and subjected to relevant staining. H&E staining expressed the formation of blood vessel-like structures in both groups (Figure 5B). Surprisingly, the PL-dECM group exhibited an ideal pulp-like matrix characterized by a relatively dense eosin-stained matrix with numerous ordered nuclei and a predentin-like structure close to the tooth section. Immunohistochemical staining further revealed that PLDSCs in the PL-dECM group possessed higher protein expression levels of DSPP and DMP-1, suggesting that PL-dECM positively affects PLDSCs *in vivo* (Figure 5C and D). For angiogenic markers, the number of PLDSCs expressing VEGF was slightly higher in PL-dECM group compared to the type I collagen group (Figure 5E). In addition, no significant difference was shown in H&E staining of essential organs (kidney, liver, lung, spleen) between nude mice that received or did not receive transplantation, indicating that PL-dECM exhibited good biocompatibility, making it suitable for tissue regeneration (Figure 5F).

## Discussion

The extracellular matrix is a dynamic and intricate network containing abundant bioactive molecules, functioning as an optimal microenvironment [18, 43]. The dECM is generated by removing cellular components and preserving the ECM through decellularized methods. This aims to simulate the natural environment and serve as a reservoir of physicochemical signals and bioactive molecules to support the survival, proliferation and differentiation of MSCs, which is considered a suitable choice for tissue engineering [9, 44, 45]. Due to the requirement of pulp regeneration and the current challenges related to insufficient sources and ethical concerns of decellularized dental pulp in clinical applications, a new approach involving dECM from periapical lesions was proposed.

The current research on extracellular matrix mainly focuses on tissue development and regeneration [44]. There are also studies on dECM derived from disease tissues, exhibiting a close correlation between the effects of dECM and its characteristics of the disease [42, 46–49]. Similarly, chronic apical periodontitis is an inflammation-related bone disease resulting from root canal infection, where various immune and non-immune cells interact to balance the inflammatory response and promote tissue restoration [28]. The osteogenic potential of MSCs increases in the inflammatory state of apical periodontitis [27]. The periapical lesions analyzed in this study were obtained from teeth that had completed root canal treatment and underwent periapical surgery. It is speculated that periapical lesions are generally in the repair stage, and PL-dECM is also considered a scaffold that tends to make a regenerative microenvironment. Therefore, we tried to perform decellularized treatment on the periapical lesion removed from the periapical surgery, expecting to obtain a new source of dECM that could provide a preferable microenvironment for tissue regeneration and meet the need for autologous transplantation.

Decellularization methods, such as hypertonic/hypotonic solution treatment, freeze-thaw cycle treatment and surfactant treatment, have been employed in various decellularized processes [50–52]. In this study, the surfactant treatment method using 0.2% SDS solution was chosen based on the characteristics of periapical lesions and in reference to related pulp tissue decellularization methods [17, 18, 53–55]. Our results showed that the PL-dECM samples met the standard requirements for decellularization treatment, exhibited a loose and porous structure, and retained the main ingredients of the extracellular matrix [41].

Through proteomic analysis, we further analyzed the components and related functions of PL-dECM. According to DIA proteomic sequencing, there was high expression of protein synthesis and cell adhesion in PL-dECM. In terms of cell adhesion, focal adhesion and calcium binding were highly expressed, among which annexins family, such as ANXA5 and ANXA2, play an essential role in biomineralization and osteogenic differentiation [56–61]. Subsequent analysis of the ECM components demonstrated that the most abundant protein in PL-dECM was collagen, including type I, XII, VI and III, which were essential elements of dental pulp matrix and bone matrix, and played active roles in biomineralization, intercellular communication and cellular attachment [62–72]. The second abundant component in PL-dECM was the glycoproteins’ part, compromising fibrinogen, fibrillin-1 and periostin, which were equipped for promoting tissue regeneration as reported [73–76]. Consistent with the above findings, other proteome analyses of the decellularized placenta, decellularized tendon, decellularized matrix derived from small intestinal submucosa and decellularized dental pulp showed that they not only contained a substantial presence of ECM and adhesion-related proteins but also retained an abundance of tissue-specific proteins, which are crucial for tissue engineering [77–81]. Hence, it could be inferred that the PL-dECM contains numerous bioactive ingredients that exert positive cellular responses, warranting further discussion. Consequently, we proceeded to conduct the following cellular and animal experiments.

Different from many reports on the use of DPSCs for pulp regeneration, and considering the difficulties in obtaining homologous MSCs for clinical application, we selected PLDSCs with multi-lineage differentiation ability, which enriches perspective for the source and subsequent application of maxillofacial related stem cells. The single-cell sequencing of apical periodontitis identified PLDSCs as four different subclusters: osteolineage, endothelial, inflammatory and neurological MSCs, indicating more advantageous in angiogenic and neural differentiation to serve as seed cells for tissue regeneration [27, 38].

The biological activity of PL-dECM *in vitro* was firstly evaluated utilizing PLDSCs. When PLDSCs co-cultured with PL-dECM leachate which simulated the initial cell culture, it showed no significant effect on the proliferative or migrative capacity, indicating almost no residue of SDS solution or DNase in PL-dECM, thus confirming the decellularized approach's rationality.

While PL-dECM conditioned medium, a soluble state of PL-dECM, which was obtained through complete pepsin digestion of PL-dECM, showed a solid dose-dependent promoting effect on improving the proliferative and migrative abilities of PLDSCs, indicating the presence of soluble bioactive ingredients. The PL-dECM conditioned medium simulated the process when cells secreted matrix metalloproteinase to resolve extracellular matrix for tissue reconstruction and remodeling [82, 83]. Proteomic analysis confirmed that the PL-dECM has higher expression of extracellular matrix components that promote cell proliferation and migration. The findings were validated by additional studies utilizing soluble dECM from different origins [18, 84–86].

Due to the obstacle to collecting a large amount of conditioned medium and the difficulty of directly observing the interaction between cells and ECM, PL-dECM was fabricated into 100-μm thick slices for cell culture. It's an effective method for recellularization to facilitate precise observation of ECM–cell interactions, which has been used in many studies [87–89]. Type I collagen, commonly used for assessing pulp regeneration, was selected as a control to compare with PL-dECM [90, 91]. Live/dead assay and immunocytochemistry staining revealed the regulatory ability of PL-dECM slices on PLDSCs compared to type I collagen. On the third day of culture, the odontogenic/osteogenic differentiation markers were upregulated in the PL-dECM slices group. After culturing for 7 days, PLDSCs in PL-dECM slices exhibited higher expression levels of DSPP, DMP-1 and VEGF, with limited improvement in the expression of Runx2 compared to the control group on mRNA level, indicating a greater inclination towards differentiation into odontoblasts. However, as PL-dECM slices are unable to fully cover the bottom dish, some cells would inevitably come into contact with the bottom, which means the contact conditions could not be controlled consistently in repeated experiments of different batches, making it difficult to stably evaluate the results. Therefore, the results of immunocytochemistry staining are more accurate and intuitive and the experimental design of PL-dECM slides should be improved later. Additionally, hydrolytic and enzymatic degradation assessment demonstrated that the structure of PL-dECM exhibits greater stability, allowing to maintain its original integrity in prolonged exposure to water or enzymes and facilitating long-lasting support for cells.

Subsequently, PLDSCs and scaffolds (PL-dECM in the experimental group and type I collagen in the control group) were placed into treated tooth sections to simulate pulp regeneration *in vivo*. The results were consistent with those of the *in vitro* experiments, which revealed the positive regulatory effect of PL-dECM on the odontogenic and angiogenic differentiation. Further investigation of the cellular immune response to PL-dECM, the degradation rate of PL-dECM in the root canals and its promotive ability for pulp regeneration should be conducted in large animal models. The specific mechanisms of ECM–cell interactions, the signaling pathways involved in cell adhesion (e.g. the annexins family), the regulatory function of highly expressed components and the role of cryptic peptides released during tissue remodeling require further study. Besides, considering the varying developmental stages of apical periodontitis, animal experiments would be considered to elucidate the regulatory effects of extracellular matrix derived from apical periodontitis on different states.

## Conclusion

The dECM derived from periapical lesion was successfully constructed while retaining the main components of the ECM. It resulted in a microscopically loose and porous structure with great cell affinity. The conditioned medium of PL-dECM was highly nutritious, promoting the proliferation and migration ability of PLDSCs. Additionally, PL-dECM slices were able to exert a positive effect on the odontogenic and angiogenetic differentiation of PLDSCs. The subcutaneous transplantation model in nude mice yielded similar results, suggesting that the dECM from periapical lesion holds promise as a bioactive scaffold. The PL-dECM is anticipated to be utilized for *in situ* transplantation in future clinical applications to support pulp regeneration.

## Supplementary Material

rbae050_Supplementary_Data

## Data Availability

Data will be made available on request.
