# Supplementary material for: Periapical lesion-derived decellularized extracellular matrix as a potential solution for regenerative endodontics
Source: Regen Biomater. 2024 May 7;11:rbae050. doi: 10.1093/rb/rbae050 (PMC11170217; doi:10.1093/rb/rbae050)
Supplement: rbae050_Supplementary_Data [file rbae050_supplementary_data.docx]

Supplementary Information

Periapical lesion-derived decellularized extracellular matrix as a potential solution for regenerative endodontic

Nan Hu ^1,4,5,6,7,9,^^♯^, Ruixue Jiang ^2,4,5,6,7,8,♯^, Yuwei Deng ^2,4,5,6,7,8^, Weiping Li ^3,4,5,6,7,9,10^, Wentao Jiang ^1,4,5,6,7,9^, Ningwei Xu^1,4,5,6,7,9^, Jia Wang ^1,4,5,6,7,9,^*, Jin Wen ^2,4,5,6,7,8,^* and Shensheng Gu ^1,4,5,6,7,9,^*

^1^ Department of Endodontics, Shanghai Ninth People’s Hospital, Shanghai Jiao Tong University School of Medicine, Zhizaoju Road No.639, Shanghai, 200011, China;

^2^ Department of Prosthodontics, Shanghai Ninth People’s Hospital, Shanghai Jiao Tong University School of Medicine, Zhizaoju Road No.639, Shanghai, 200011, China;

^3^ Department of Oral and Maxillofacial-Head and Neck Oncology, Shanghai Ninth People’s Hospital, Shanghai Jiao Tong University School of Medicine, Zhizaoju Road No.639, Shanghai, 200011, China;

^4^ College of Stomatology, Shanghai Jiao Tong University, Yanqiao Road No.390, Shanghai, 200125, China;

^5^ National Center for Stomatology, Zhizaoju Road No.639, Shanghai, 200011, China;

^6^ National Clinical Research Center for Oral Diseases, Zhizaoju Road No.639, Shanghai, 200011, China;

^7^ Shanghai Key Laboratory of Stomatology, Yanqiao Road No.390, Shanghai, 200125, China;

^8^ Shanghai Engineering Research Center of Advanced Dental Technology and Materials, Yanqiao Road No.390, Shanghai, 200125, China;

^9^ Shanghai Research Institute of Stomatology, Zhizaoju Road No.639, Shanghai, 200011, China

^10^ Shanghai Center of Head and Neck Oncology Clinical and Translational Science, Zhizaoju Road No.639, Shanghai, 200011, China

***** Corresponding authors

Email address: [wj2m9h@126.com](mailto:wj2m9h@126.com) (J. Wang), [echomet@126.com](mailto:echomet@126.com) (W. Jin), [gss2m9h@163.com](mailto:gss2m9h@163.com) (S. Gu).

^♯^ The authors contribute equally to this work.

1.
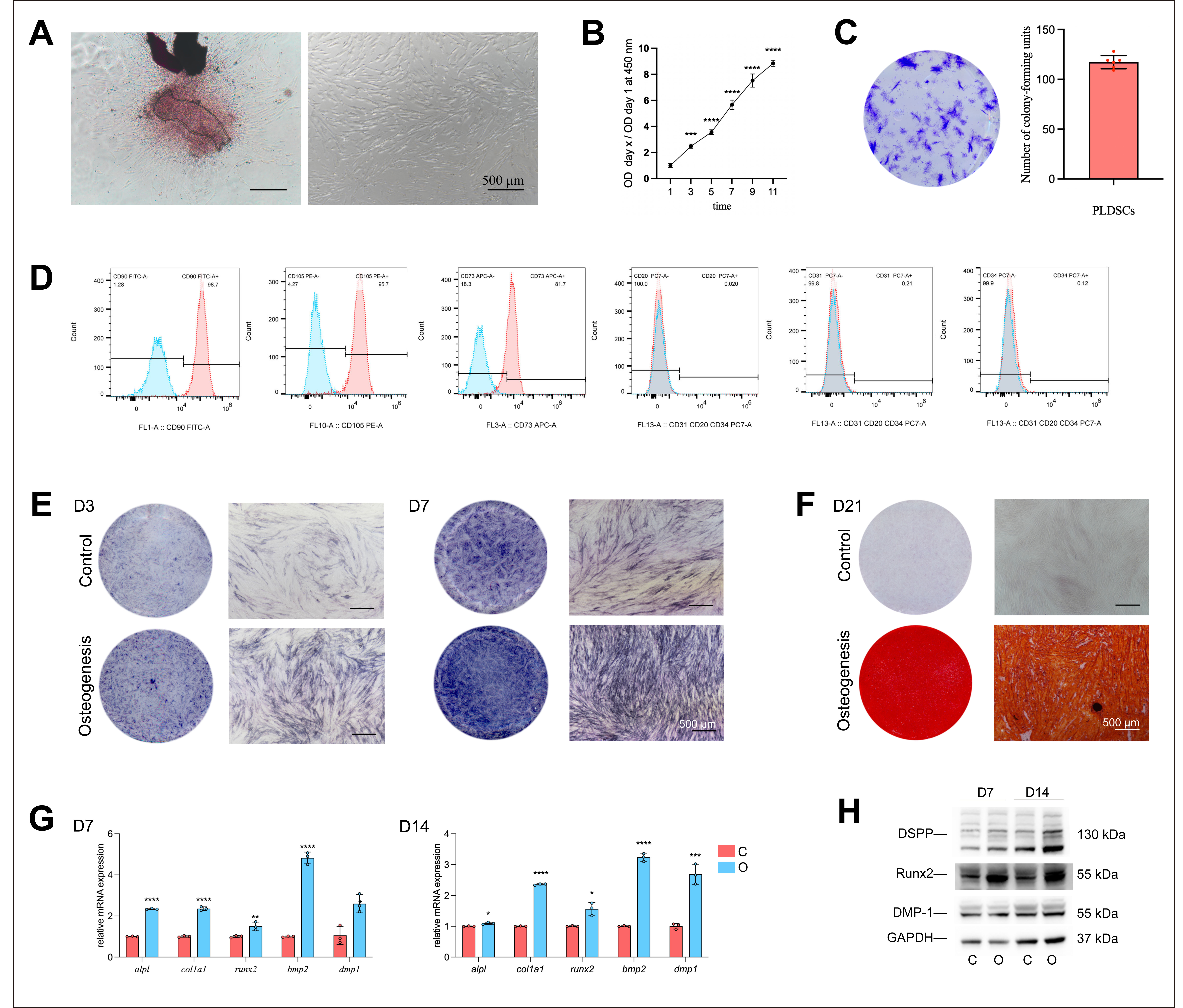
Supplementary Figure

Fig. S1. The isolation, culture, and characterization of periapical lesion-derived stem cells (PLDSCs). (A) The primary outgrowth and culture of PLDSCs; (B) Growth curve of PLDSCs from day one to eleven; (C) Colony-forming test of PLDSCs; (D) Flow cytometry assay of surface markers of PLDSCs; (E) Alkaline phosphatase staining after three days and seven days of osteogenic induction; (F) Alizarin red staining after 21 days of osteogenic induction, scale bar: 500 μm; (G, H) Relative expression of osteogenesis/odontogenesis-related genes at the mRNA and protein levels after seven days and fourteen days of osteogenesis induction (*:p<0.05, **:p<0.01, ***: p<0.001, ***: p<0.0001 as compared with the control group; C: control group; O: osteogenic group);

Fig. S2. Gross photography on the preparation of PL-dECM leachate and conditioned medium;


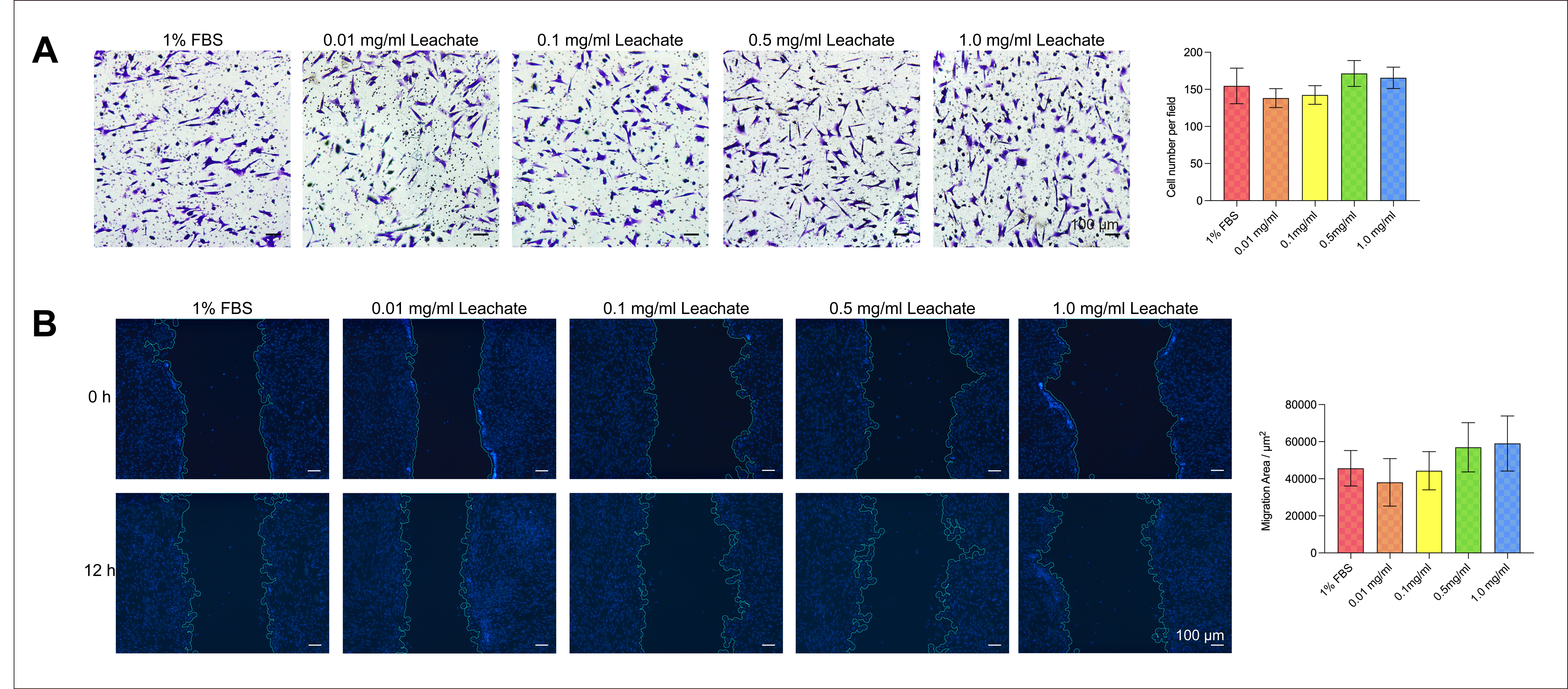


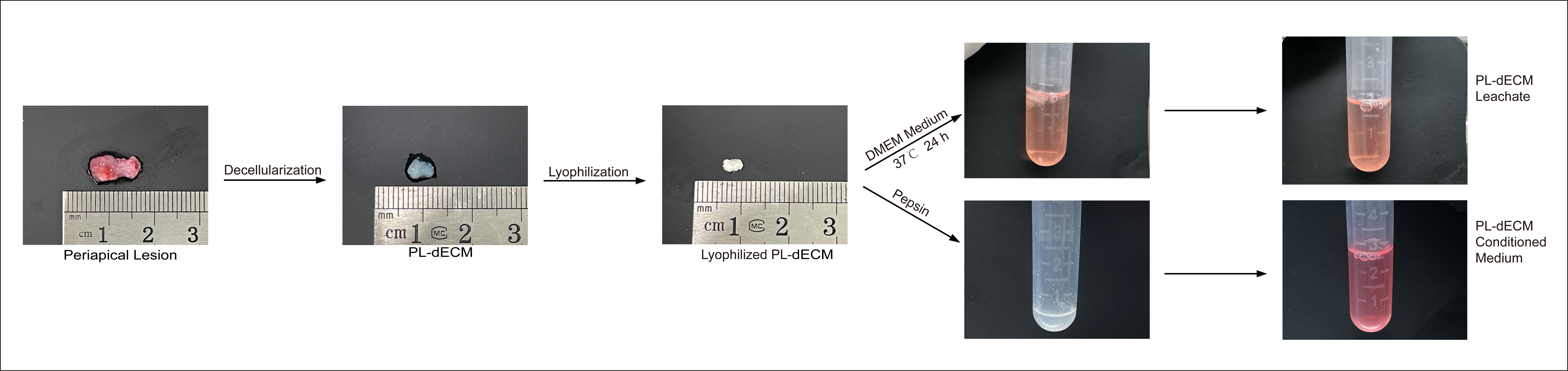
Fig. S3. Transwell assay (A) and wound healing assay (B) showed PL-dECM leachate has almost no obvious effect on the cellular migration ability, scale bar: 100 μm, Leachate: PL-dECM leachate;


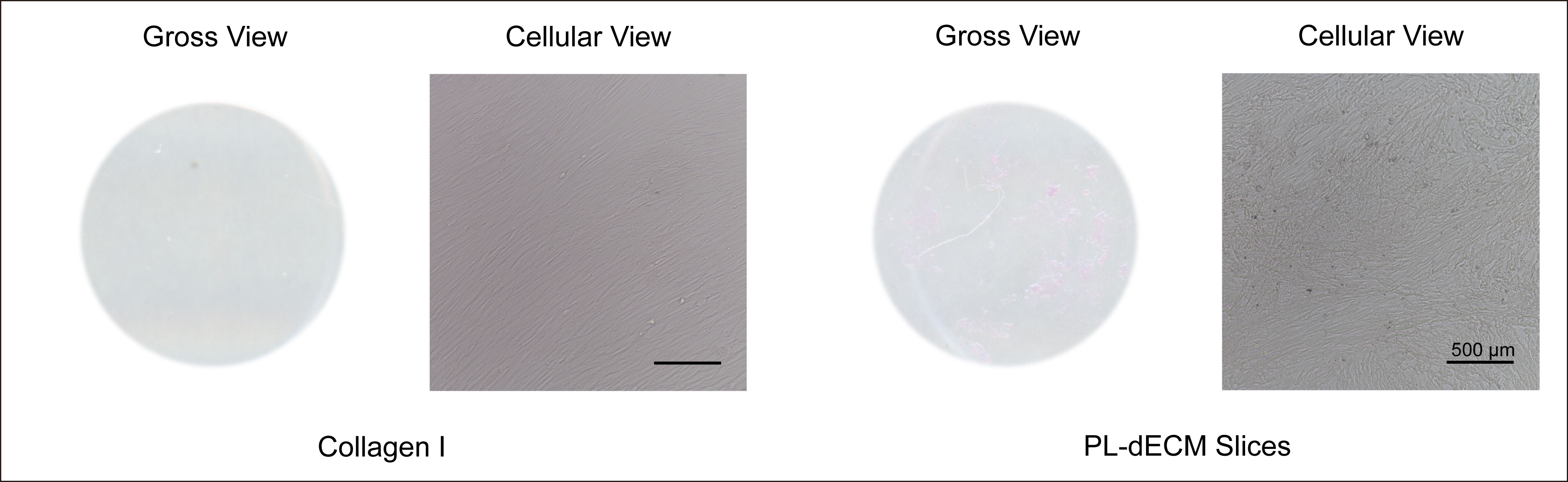


Fig. S4. The preparation of collagen I and PL-dECM slices on the bottom of the petri dish (Grow view and cellular view), scale bar: 500 μm;


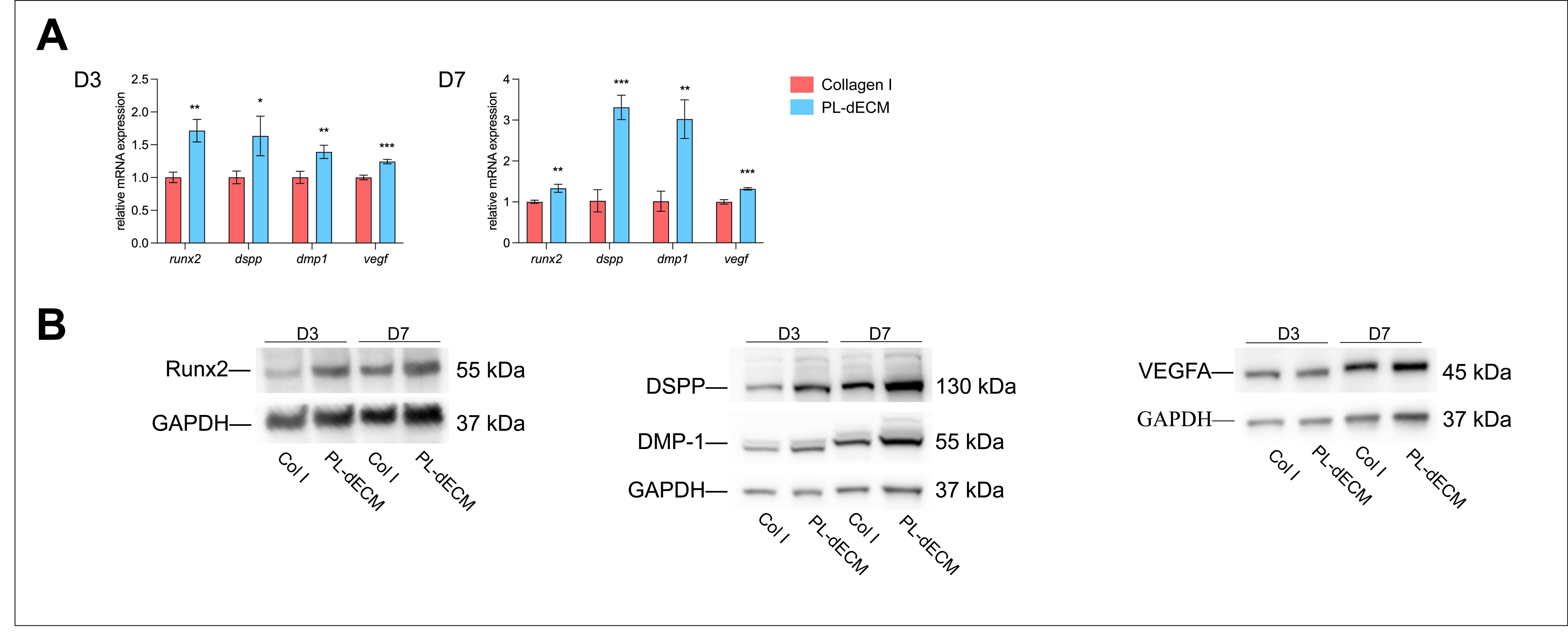
Fig. S5. Expression of odontogenic and angiogenic differentiation-related markers at the mRNA (A) and protein (B) levels after PLDSCs co-culturing with type I collagen (Col I) or PL-dECM slices (PL-dECM) for three days and seven days (*:p<0.05, **:p<0.01, ***: p<0.001 as compared with the type I collagen group);


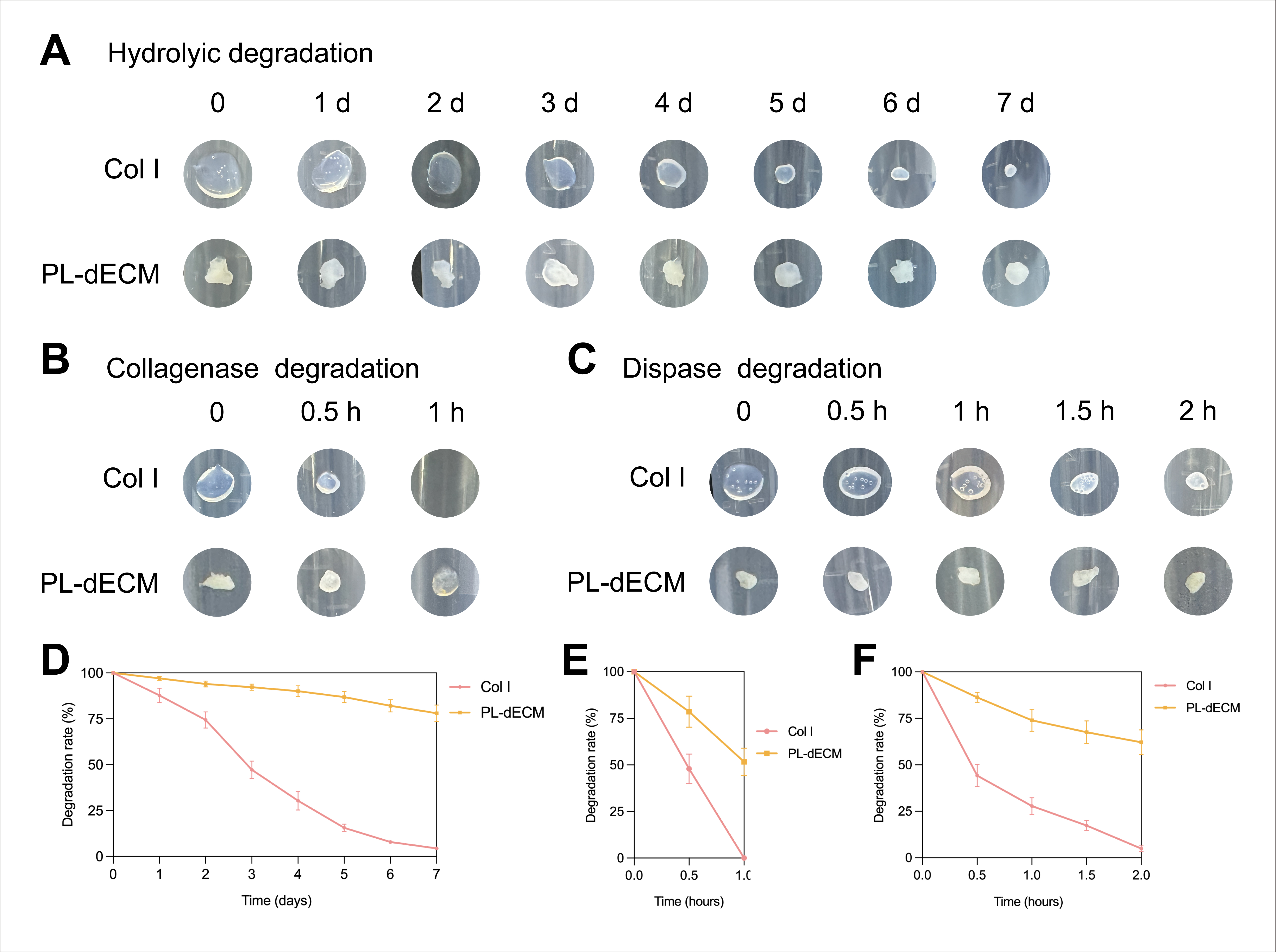


Fig. S6. (A-C) Images of type I collagen and PL-dECM for hydrolytic (A) and enzymatic (B, C) degradation. (D-E) Degradation curves of type I collagen and PL-dECM for hydrolytic (D) and enzymatic (E, F) degradation.


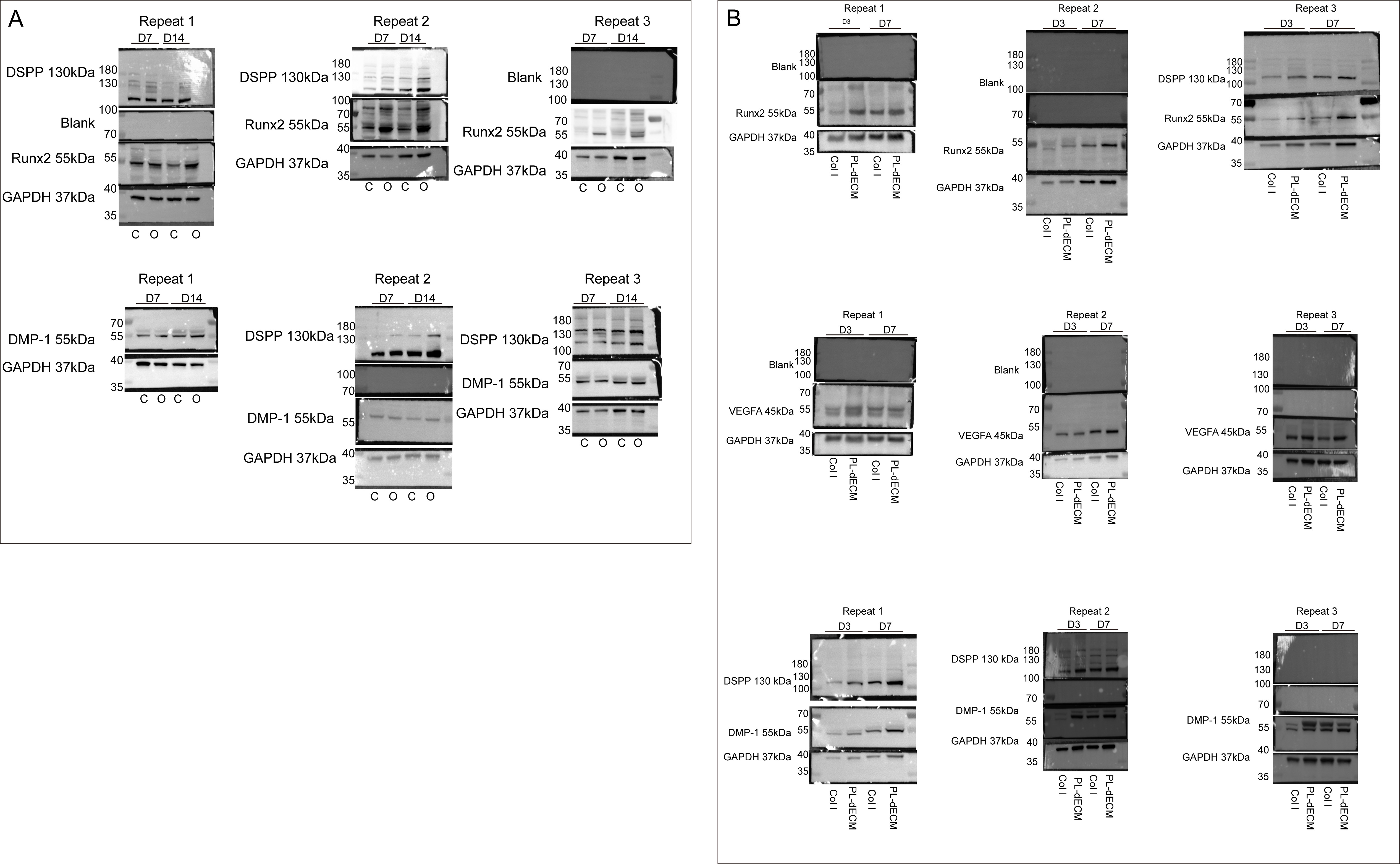


Fig. S7. Original western blot for three repeats: (A) Original western blot of the expression of osteogenesis/ odontogenesis-related genes of PLDSCs in the control group (C) and osteogenesis induction group (O)；(B) Original western blot of the expression of odontogenesis- and angiogenesis-related genes of PLDSCs culturing on collagen I (Col I) or PL-dECM slices (PL-dECM);

1. Supplementary Table

Supplementary Table 1. Primers sequences of target genes for RT-qPCR;

| Primers | Forward sequence  (5’-3’) | Reverse sequence  (5’-3’) |
| --- | --- | --- |
| ACTB | TGGCACCCAGCACAATGAA | CTAAGTCATAGTCCGCCTAGAAGCA |
| ALPL | CCTTGTAGCCAGGCCCATTG | GGACCATTCCCACGTCTTCAC |
| COL1A1 | CCAGAAGAACTGGTACATCAGCAA | CGCCATACTCGAACTGGAATC |
| Runx2 | CACTGGCGCTGCAACAAGA | CATTCCGGAGCTCAGCAGAATAA |
| BMP2 | GTCCTGAGCGAGTTCGAGTT | AGTGCCTGCGATACAGGTCT |
| DSPP | AGTGACAGCCAGAGCAAG | CCTATCCCATTACCAAACT |
| DMP-1 | TTATGGCACAGTCAGTTG | GGTGATGTTTATGGGAGT |
| VEGF | AGGGCAGAATCATCACGAAGT | AGGGTCTCGATTGGATGGCA |
|  |  |  |

Supplementary Table 2. Antibodies of target proteins for western blot;

| Antibody | Dilution | Supplier |
| --- | --- | --- |
| Primary antibody | | |
| Anti-Mouse GAPDH | 1:50000 | Proteintech, China |
| Anti-Mouse DSPP | 1:400 | Santa Cruz, USA |
| Anti-Mouse DMP-1 | 1:400 | Santa Cruz, USA |
| Anti-Rabbit Runx2 | 1:1000 | Abclonal, China |
| Anti-Rabbit VEGFA | 1:1000 | Proteintech, China |
| Secondary antibody | | |
| HRP-conjugated Goat Anti-Mouse IgG(H+L) | 1:5000 | Proteintech, China |
| HRP-conjugated Goat Anti-Rabbit IgG(H+L) | 1:10000 | Proteintech, China |

Supplementary Table 3. Antibodies of target proteins for immunofluorescent staining and immunohistochemical staining;

| Antibody | Dilution | Supplier |
| --- | --- | --- |
| Primary antibody |  |  |
| Anti-Rabbit Collagen Ⅰ | 1:200 | CST, USA |
| Anti-Rabbit Collagen Ⅲ | 1:250 | Abcam, USA |
| Anti-Rabbit Fibronectin | 1:100 | Beyotime, China |
| Anti-Rabbit Laminin | 1:100 | Bioss, China |
| Anti-Mouse DSPP | 1:100 | Santa Cruz, USA |
| Anti-Mouse DMP-1 | 1:100 | Santa Cruz, USA |
| Anti-Rabbit Runx2 | 1:100 | Abclonal, China |
| Anti-Mouse VEGF | 1:50 | Invitrogen, USA |
| Secondary antibody |  |  |
| Alexa Fluor 555-labeled Donkey Anti-Mouse IgG(H+L) | 1:500 | Beyotime, China |
| Alexa Fluor 555-labeled Donkey Anti-Rabbit IgG(H+L) | 1:500 | Beyotime, China |
| FITC-labeled Goat Anti-Rabbit IgG (H+L) | 1:500 | Beyotime, China |
| HRP-conjugated Goat Anti-Mouse IgG(H+L) | 1:50 | Beyotime, China |
| HRP-conjugated Goat Anti-Rabbit IgG(H+L) | 1:50 | Beyotime, China |

Supplementary Table 4

| **Accession** | **Gene Name** | **Description** | **MW [kDa]** | **Cscore** | **Peptides** | **Unique peptides** | **A1** | **A2** | **A3** |
| --- | --- | --- | --- | --- | --- | --- | --- | --- | --- |
| A0A075B6I0 | IGLV8-61 | Immunoglobulin lambda variable 8-61 | 12.81437 | 51.801697 | 2 | 2 | 5969.18555 | 516.29657 | 368.857483 |
| A0A075B6J9 | IGLV2-18 | Immunoglobulin lambda variable 2-18 | 12.4117 | 35.64009 | 1 | 1 | 32.3722992 | 101.774536 |  |
| A0A075B6K4 | IGLV3-10 | Immunoglobulin lambda variable 3-10 | 12.44085 | 46.537132 | 2 | 1 | 117.707695 | 104.743462 | 52.2798157 |
| A0A075B6K5 | IGLV3-9 | Immunoglobulin lambda variable 3-9 | 12.33171 | 43.862465 | 1 | 1 | 98.6705322 | 194.288452 | 85.0109711 |
| A0A075B6P5 | IGKV2-28 | Immunoglobulin kappa variable 2-28 | 12.95674 | 51.44963 | 1 | 1 | 729.813721 |  |  |
| A0A075B6R9 | IGKV2D-24 | Probable non-functional immunoglobulin kappa variable 2D-24 | 13.07893 | 50.511753 | 1 | 1 | 178.417526 | 257.819794 | 118.236244 |
| A0A075B6S2 | IGKV2D-29 | Immunoglobulin kappa variable 2D-29 | 13.14312 | 49.907986 | 3 | 1 | 1162.36926 | 1298.61169 | 417.665497 |
| A0A075B6S5 | IGKV1-27 | Immunoglobulin kappa variable 1-27 | 12.71151 | 50.860344 | 1 | 1 | 1119.73169 | 159.494736 | 47.542099 |
| A0A087WSX0 | IGLV5-45 | Immunoglobulin lambda variable 5-45 | 13.16178 | 41.551403 | 1 | 1 | 26.8659706 | 48.9451981 | 26.3841457 |
| A0A0A0MRZ8 | IGKV3D-11 | Immunoglobulin kappa variable 3D-11 | 12.62524 | 53.539 | 1 | 1 | 2584.229 | 2974.61963 | 1609.59058 |
| A0A0B4J1V0 | IGHV3-15 | Immunoglobulin heavy variable 3-15 | 12.92578 | 46.767273 | 4 | 3 | 45.824604 | 257.090607 | 29.8821659 |
| A0A0B4J1X5 | IGHV3-74 | Immunoglobulin heavy variable 3-74 | 12.83961 | 49.16128 | 1 | 1 | 715.076538 | 18.1142921 |  |
| A0A0B4J1Y8 | IGLV9-49 | Immunoglobulin lambda variable 9-49 | 13.02367 | 51.08173 | 1 | 1 | 143.663605 | 79.2609024 | 13.1170874 |
| A0A0B4J2F0 | PIGBOS1 | Protein PIGBOS1 | 6.31338 | 43.986496 | 1 | 1 | 32.2113724 | 42.6010971 | 7.90515614 |
| A0A0C4DH25 | IGKV3D-20 | Immunoglobulin kappa variable 3D-20 | 12.51507 | 48.666695 | 1 | 1 | 1111.33911 | 336.348724 | 213.520721 |
| A0A0C4DH29 | IGHV1-3 | Immunoglobulin heavy variable 1-3 | 13.00772 | 35.27772 | 1 | 1 | 43.5153389 | 19.9735641 | 13.8849106 |
| A0A0C4DH31 | IGHV1-18 | Immunoglobulin heavy variable 1-18 | 12.82043 | 42.35371 | 1 | 1 | 135.669678 |  |  |
| A0A0C4DH36 | IGHV3-38 | Probable non-functional immunoglobulin heavy variable 3-38 | 12.75854 | 45.403698 | 1 | 1 | 151.638931 |  |  |
| A0A0C4DH38 | IGHV5-51 | Immunoglobulin heavy variable 5-51 | 12.67455 | 50.8377 | 3 | 2 | 467.947266 | 382.900269 | 105.467583 |
| A0A0J9YX35 | IGHV3-64D | Immunoglobulin heavy variable 3-64D | 12.82257 | 36.66756 | 1 | 1 | 91.949295 |  | 16.1721001 |
| A0A1B0GVG4 | CCDC194 | Coiled-coil domain-containing protein 194 | 24.95415 | 46.05283 | 1 | 1 | 27.1443386 |  |  |
| A0AV96 | RBM47 | RNA-binding protein 47 | 64.09908 | 44.76328 | 1 | 1 | 59.8580894 | 19.0547695 | 10.1678076 |
| A0AVT1 | UBA6 | Ubiquitin-like modifier-activating enzyme 6 | 117.96997 | 45.964344 | 1 | 1 | 29.0334511 | 7.72458172 | 7.55187511 |
| A0FGR8 | ESYT2 | Extended synaptotagmin-2 | 102.35748 | 52.37795 | 10 | 10 | 227.254913 | 88.9556885 | 85.60112 |
| A1L0T0 | ILVBL | 2-hydroxyacyl-CoA lyase 2 | 67.86774 | 48.605106 | 3 | 3 | 106.547569 | 66.8448181 | 53.4746628 |
| A1X283 | SH3PXD2B | SH3 and PX domain-containing protein 2B | 101.57909 | 53.08716 | 5 | 5 | 145.727936 | 50.0191307 | 82.6249695 |
| A2A288 | ZC3H12D | Probable ribonuclease ZC3H12D | 58.07785 | 40.7754 | 1 | 1 | 11.7598276 | 9.26430511 | 6.15208721 |
| A2RRP1 | NBAS | NBAS subunit of NRZ tethering complex | 268.57131 | 42.79931 | 4 | 4 | 66.3647919 | 43.2000389 | 42.5221939 |
| A2RTX5 | TARS3 | Threonine--tRNA ligase 2, cytoplasmic | 92.64555 | 44.058357 | 3 | 1 | 119.405396 | 21.678606 | 12.2996893 |
| A3KMH1 | VWA8 | von Willebrand factor A domain-containing protein 8 | 214.82445 | 51.569157 | 5 | 5 | 21.9638653 | 35.1073151 | 19.2801323 |
| A4D1P6 | WDR91 | WD repeat-containing protein 91 | 83.3444 | 39.591915 | 1 | 1 | 65.3109741 | 76.0227051 | 85.2294312 |
| A5PLL7 | PEDS1 | Plasmanylethanolamine desaturase | 31.13497 | 40.536957 | 1 | 1 | 23.8193798 | 12.003068 | 13.2936096 |
| A5YKK6 | CNOT1 | CCR4-NOT transcription complex subunit 1 | 266.93887 | 52.38692 | 5 | 5 | 16.863842 | 15.620245 | 15.5022564 |
| A6NE01 | FAM186A | Protein FAM186A | 262.75819 | 23.973719 | 1 | 1 | 4473.51709 | 2134.30151 | 1435.26147 |
| A6NFQ2 | TCAF2 | TRPM8 channel-associated factor 2 | 100.90605 | 54.466038 | 3 | 3 | 100.380775 | 17.9065533 | 22.5890579 |
| A6NGU5 | GGT3P | Putative glutathione hydrolase 3 proenzyme | 61.50157 | 51.708637 | 3 | 2 | 59.8468323 | 133.653778 | 105.88414 |
| A6NHL2 | TUBAL3 | Tubulin alpha chain-like 3 | 49.90873 | 44.664547 | 1 | 1 | 61.2896767 | 10.2708092 | 15.1936121 |
| A6NHR9 | SMCHD1 | Structural maintenance of chromosomes flexible hinge domain-containing protein 1 | 226.37357 | 52.03044 | 12 | 12 | 49.7101173 | 35.3188858 | 30.6742344 |
| A6NI72 | NCF1B | Putative neutrophil cytosol factor 1B | 44.81701 | 46.53317 | 1 | 1 | 32.9292793 | 7.86876869 | 4.11921024 |
| A6NI79 | CCDC69 | Coiled-coil domain-containing protein 69 | 34.79626 | 38.85166 | 1 | 1 | 8.42710495 | 18.4492092 | 15.0441637 |
| A6NJI1 | C11orf86 | Uncharacterized protein C11orf86 | 13.17174 | 33.126965 | 1 | 1 | 277.598724 | 182.574249 | 132.428848 |
| A6NMZ7 | COL6A6 | Collagen alpha-6(VI) chain | 247.17335 | 38.031933 | 1 | 1 |  | 55.9955025 |  |
| A6NNC1 |  | Putative POM121-like protein 1-like | 94.05836 | 24.860125 | 1 | 1 | 1295.89905 | 2592.51465 | 2693.11987 |
| A7KAX9 | ARHGAP32 | Rho GTPase-activating protein 32 | 230.52901 | 43.576355 | 1 | 1 | 34.145401 | 5.55358315 | 10.4893103 |
| A8K2U0 | A2ML1 | Alpha-2-macroglobulin-like protein 1 | 161.10652 | 48.37587 | 12 | 12 | 76.4240189 | 3.59178853 | 20.0311127 |
| A8MWD9 | SNRPGP15 | Putative small nuclear ribonucleoprotein G-like protein 15 | 8.54404 | 48.924667 | 2 | 2 | 165.445816 | 128.770874 | 115.222649 |
| A8MYU2 | KCNU1 | Potassium channel subfamily U member 1 | 129.54343 | 24.614216 | 1 | 1 | 81.8907318 | 19.3645134 | 17.8742161 |
| B0I1T2 | MYO1G | Unconventional myosin-Ig | 116.44207 | 52.46531 | 15 | 13 | 135.888458 | 194.757309 | 153.524307 |
| B3KS81 | SRRM5 | Serine/arginine repetitive matrix protein 5 | 80.35482 | 35.057922 | 1 | 1 | 40.8949699 | 75.0242004 | 128.207382 |
| B5ME19 | EIF3CL | Eukaryotic translation initiation factor 3 subunit C-like protein | 105.47291 | 55.899284 | 14 | 14 | 241.421036 | 119.71656 | 104.589348 |
| B7ZAQ6 | GPR89A | Golgi pH regulator A | 52.91696 | 49.174976 | 4 | 4 | 47.9597778 | 46.7160339 | 31.892313 |
| B9A064 | IGLL5 | Immunoglobulin lambda-like polypeptide 5 | 23.06324 | 51.001114 | 4 | 1 | 3893.79468 | 9262.75879 | 2513.43335 |
| E9PAV3 | NACA | Nascent polypeptide-associated complex subunit alpha, muscle-specific form | 205.42152 | 47.202274 | 5 | 4 | 304.429199 | 117.768593 | 87.8263016 |
| E9PRG8 | C11orf98 | Uncharacterized protein C11orf98 | 14.23372 | 46.33285 | 1 | 1 | 65.1670456 | 19.7796898 | 15.9349899 |
| G2XKQ0 | SUMO1P1 | Small ubiquitin-related modifier 5 | 11.52604 | 45.209026 | 1 | 1 | 40.5214806 | 20.0380211 | 16.7121296 |
| O00116 | AGPS | Alkyldihydroxyacetonephosphate synthase, peroxisomal | 72.91181 | 39.120823 | 1 | 1 | 15.7737389 | 8.65409279 | 9.34772587 |
| O00139 | KIF2A | Kinesin-like protein KIF2A | 79.95454 | 52.814274 | 2 | 2 | 81.0261765 | 45.2717361 | 31.7469845 |
| O00142 | TK2 | Thymidine kinase 2, mitochondrial | 31.00477 | 49.355682 | 1 | 1 | 179.28241 | 302.567871 | 159.601807 |
| O00151 | PDLIM1 | PDZ and LIM domain protein 1 | 36.07171 | 54.073757 | 9 | 9 | 311.151123 | 83.1719589 | 47.2784081 |
| O00159 | MYO1C | Unconventional myosin-Ic | 121.68167 | 55.42756 | 30 | 30 | 339.589417 | 202.763611 | 198.806351 |
| O00160 | MYO1F | Unconventional myosin-If | 124.84428 | 49.28655 | 6 | 3 | 137.554138 | 60.640152 | 94.791153 |
| O00161 | SNAP23 | Synaptosomal-associated protein 23 | 23.35404 | 47.12373 | 5 | 5 | 108.792702 | 152.01947 | 105.260963 |
| O00170 | AIP | AH receptor-interacting protein | 37.66406 | 45.780758 | 2 | 2 | 44.3067818 | 43.2540207 | 37.423954 |
| O00178 | GTPBP1 | GTP-binding protein 1 | 72.45381 | 41.317917 | 1 | 1 | 30.3932781 | 15.9937668 | 21.7180538 |
| O00182 | LGALS9 | Galectin-9 | 39.51817 | 51.532246 | 2 | 1 | 35.6089172 | 34.9465637 | 17.1026211 |
| O00193 | SMAP | Small acidic protein | 20.33252 | 42.55438 | 1 | 1 | 70.875 | 22.0263653 | 13.9465046 |
| O00203 | AP3B1 | AP-3 complex subunit beta-1 | 121.32017 | 53.68936 | 14 | 13 | 162.924057 | 94.0177765 | 66.8330841 |
| O00204 | SULT2B1 | Sulfotransferase 2B1 | 41.30771 | 44.694126 | 1 | 1 | 75.6587982 |  | 13.9254656 |
| O00214 | LGALS8 | Galectin-8 | 35.80811 | 47.582516 | 3 | 3 | 68.6217117 | 18.857336 | 21.626503 |
| O00231 | PSMD11 | 26S proteasome non-ATPase regulatory subunit 11 | 47.46377 | 51.962337 | 9 | 9 | 166.078827 | 45.4956818 | 45.1077881 |
| O00232 | PSMD12 | 26S proteasome non-ATPase regulatory subunit 12 | 52.9044 | 50.37073 | 5 | 5 | 42.6594772 | 11.5940952 | 6.93901873 |
| O00244 | ATOX1 | Copper transport protein ATOX1 | 7.40162 | 51.966137 | 1 | 1 | 40.288887 | 7.73087215 | 12.8462038 |
| O00264 | PGRMC1 | Membrane-associated progesterone receptor component 1 | 21.67115 | 46.041615 | 2 | 2 | 354.218414 | 70.013916 | 106.306625 |
| O00267 | SUPT5H | Transcription elongation factor SPT5 | 120.99957 | 47.179398 | 3 | 3 | 13.0357046 | 22.4285851 | 6.3002882 |
| O00273 | DFFA | DNA fragmentation factor subunit alpha | 36.5219 | 28.731642 | 1 | 1 | 47.2139282 | 34.6476707 | 13.4067993 |
| O00299 | CLIC1 | Chloride intracellular channel protein 1 | 26.92272 | 47.1709 | 6 | 6 | 84.8676987 | 21.0657787 | 8.63847828 |
| O00303 | EIF3F | Eukaryotic translation initiation factor 3 subunit F | 37.56385 | 49.71826 | 4 | 4 | 38.606411 | 14.5515718 | 13.7199478 |
| O00339 | MATN2 | Matrilin-2 | 106.83692 | 50.141064 | 8 | 8 | 118.453835 | 149.836105 | 45.1587791 |
| O00391 | QSOX1 | Sulfhydryl oxidase 1 | 82.57773 | 47.190712 | 1 | 1 | 56.3958817 | 31.2678452 |  |
| O00400 | SLC33A1 | Acetyl-coenzyme A transporter 1 | 60.90899 | 47.4552 | 2 | 2 | 91.8383942 | 31.1643677 | 27.8930359 |
| O00410 | IPO5 | Importin-5 | 123.62993 | 45.413483 | 8 | 7 | 136.716522 | 54.1034775 | 65.290535 |
| O00422 | SAP18 | Histone deacetylase complex subunit SAP18 | 17.56107 | 33.3052 | 1 | 1 | 35.3228416 | 13.639679 | 32.3422012 |
| O00429 | DNM1L | Dynamin-1-like protein | 81.87715 | 50.201477 | 3 | 3 | 76.5409012 | 31.5580254 | 36.0642471 |
| O00442 | RTCA | RNA 3'-terminal phosphate cyclase | 39.33673 | 27.690613 | 2 | 2 | 20.7109375 | 12.6714296 | 3.26554251 |
| O00461 | GOLIM4 | Golgi integral membrane protein 4 | 81.88031 | 53.5815 | 3 | 3 | 39.8852806 | 23.2920017 | 34.4333954 |
| O00468 | AGRN | Agrin | 217.31953 | 44.9461 | 3 | 3 | 175.281769 | 90.1076355 | 74.8721848 |
| O00469 | PLOD2 | Procollagen-lysine,2-oxoglutarate 5-dioxygenase 2 | 84.68561 | 56.567966 | 3 | 3 | 13.7535343 |  | 1.91703415 |
| O00471 | EXOC5 | Exocyst complex component 5 | 81.85287 | 41.051388 | 1 | 1 | 23.0832443 |  |  |
| O00479 | HMGN4 | High mobility group nucleosome-binding domain-containing protein 4 | 9.53891 | 53.45765 | 1 | 1 | 126.736908 | 75.5093079 | 14.1307859 |
| O00483 | NDUFA4 | Cytochrome c oxidase subunit NDUFA4 | 9.36986 | 52.55627 | 2 | 2 | 121.896797 | 51.3546448 | 36.5325203 |
| O00487 | PSMD14 | 26S proteasome non-ATPase regulatory subunit 14 | 34.57703 | 46.33536 | 1 | 1 | 196.584656 | 107.283226 | 86.8696976 |
| O00505 | KPNA3 | Importin subunit alpha-4 | 57.81092 | 47.03766 | 3 | 1 | 157.261566 | 28.1397896 | 27.9951668 |
| O00515 | LAD1 | Ladinin-1 | 57.13106 | 45.984425 | 2 | 2 | 34.7776718 |  | 19.4223537 |
| O00534 | VWA5A | von Willebrand factor A domain-containing protein 5A | 86.48926 | 45.197105 | 4 | 4 | 44.6018028 | 45.4282646 | 42.182312 |
| O00560 | SDCBP | Syntenin-1 | 32.44438 | 53.314426 | 4 | 4 | 93.852356 | 17.6366425 | 20.7198792 |
| O00567 | NOP56 | Nucleolar protein 56 | 66.04989 | 50.01691 | 13 | 13 | 126.532532 | 68.72052 | 43.2645073 |
| O00571 | DDX3X | ATP-dependent RNA helicase DDX3X | 73.24341 | 52.45795 | 16 | 4 | 830.238953 | 393.198853 | 303.65451 |
| O00592 | PODXL | Podocalyxin | 58.63525 | 42.751785 | 1 | 1 | 61.9491081 | 86.7217255 | 28.001297 |
| O00602 | FCN1 | Ficolin-1 | 35.0784 | 41.351696 | 1 | 1 | 8.93178463 | 43.2173424 | 21.1357956 |
| O00767 | SCD | Stearoyl-CoA desaturase | 41.52267 | 47.08843 | 2 | 2 | 204.593414 | 30.9270668 | 28.1459198 |

Supplementary Method

1. Flow cytometry assay

The PLDSCs were digested with trypsin and resuspended in PBS to adjust the density to 1×10^6/mL. Then the cells were incubated with CD90-FITC (BioGems, USA), CD105-PE (BioGems, USA), CD73-APC (BioGems, USA), CD31-PECY7 (Biolegend, USA), CD20-PECY7 (BioGems, USA) and CD74-PECY7 (BioGems, USA) antibodies for 45 minutes at room temperature in dark environment, resuspended in PBS buffer and then were analyzed via a Flow Cytometer (Beckman Cytoflex).

1. Cell proliferation assay

The PLDSCs were seeded in 96-well plates at a density of 4×10^3 per well, and CCK-8 experiments were performed on 1, 3, 5, 7, 9, and 11 days after cell culture. The CCK-8 working solution (CK04, Dojindo, Japan) was incubated for 30 minutes. Then, OD values at 450 nm were measured in a microplate reader, and relative OD values were calculated for each time point.

1. Colony-forming test

The PLDSCs were seeded in a six-well plate at a density of 1×10^3 cells per well. After 12 days of culture, the cells were rinsed with PBS buffer, fixed with 4% paraformaldehyde for 20 minutes, stained with 1% crystal violet staining solution for 15 minutes, and washed with ddH_2_O. Crystal violet-stained cells were observed and counted under an inverted microscope, and the colony formation rate was calculated based on the standard that >50 cells were counted as one colony.

1. Alkaline phosphatase staining test

The PLDSCs were seeded in a 24-well plate at a density of 2.5×10^4 /mL. When the cell confluence reached about 90%, the experimental group was switched to osteogenesis/odontogenesis culture medium (DMEM medium containing 10% FBS, 50ug/mL ascorbic acid, 10mM β-Glycerophosphate sodium, and 10 nmol/L dexamethasone). Alkaline phosphatase staining (Alkaline phosphatase staining kit, C3206, Beyotime, China) was performed for 3 and 7 days after further culture. Specifically, the BCIP/NBT alkaline phosphatase staining solution is prepared according to the instructions. The cells are washed with PBS, fixed with 4% paraformaldehyde for 20 minutes, stained with BCIP/NBT alkaline phosphatase staining solution for 30 minutes in a dark environment, and rinsed with ddH_2_O three times. The staining results were observed and recorded under an inverted microscope.

1. Alizarin red staining

PLDSCs were seeded in 24-well plates at 2.5×10^4/mL. When the cell confluence reached about 90%, the experimental group was switched to an osteogenesis/odontogenesis induction solution. After 21 days of culture, the cells were rinsed with PBS three times, fixed with 4% paraformaldehyde for 20 minutes, incubated with Alizarin red staining solution (G1452, Solarbio, China) for 20 minutes, and washed with ddH_2_O. The staining results were observed and recorded under an inverted microscope.

1. Hydrolytic degradation evaluation:

The extra water in type I collagen or PL-dECM was removed using blotting paper. The weights of type I collagen and PL-dECM were measured and recorded as m_0_. Then the type I collagen and PL-dECM were placed in centrifuge tubes with 1 mL PBS solution (pH=7.4) immersed. The centrifuge tubes were positioned on a shaker at a speed of 20 rpm and maintained at 37℃ for seven days. The immersing PBS solution was replaced daily from the first to the seventh day and the weights of type I collagen and PL-dECM were measured and recorded as m_x_ for day x after wiping with blotting paper. The degradation rate of the sample on day x was calculated as $\frac{m_{0}-m_{x}}{m_{0}}\times100\%$. Additionally, the hydrolytic degradation curve of type I collagen and PL-dECM was visualized, and images of both were captured from day 1 to 7.

1. Enzymatic degradation evaluation:

Considering the abundant presence of type I collagen in PL-dECM, collagenase type I (BS032B, Biosharp, China) was used for the enzymatic degradation test. Similarly, the extra water in type I collagen or PL-dECM was removed through blotting paper. The weights of two samples were measured and recorded. Subsequently, the type I collagen and PL-dECM were placed in centrifuge tubes with 1 mL collagenase type I solution (0.2 mg/mL) immersed. The centrifuge tubes were positioned on a shaker at a speed of 20 rpm and 37℃. After incubating for 30 and 60 minutes, the weights of type I collagen and PL-dECM were measured and recorded. The enzymatic degradation curve and the images of type I collagen and PL-dECM were recorded.

To simulate the cellular process of matrix metalloproteinase secretion for extracellular matrix dissolution during tissue remodeling, dispase II (4942078001, Roche, Switzerland), a type of matrix metalloproteinase, was chosen for enzymatic test at a concentration of 0.2 mg/mL. Similar to the enzymatic degradation procedures using collagenase above, the weights of type I collagen and PL-dECM were measured and recorded after incubating for 30, 60, 90, and 120 minutes. The degradation curve and the images were recorded.
